# MXene‐Engineered Cs_2_AgBiBr_6_ Perovskite Solar Cells: Rational Screening and Interfacial Dynamics for Lead‐Free Photovoltaics

**DOI:** 10.1002/advs.202506567

**Published:** 2025-06-29

**Authors:** Lin Yang, Tianfang Zheng, Ziyan Liu, Naoyuki Shibayama, Peng Li, Jiangang Ma, Xintong Zhang, Hancheng Zhu, Xiao‐Feng Wang, Haiyang Xu, Yichun Liu

**Affiliations:** ^1^ State Key Laboratory of Integrated Optoelectronics Key Laboratory for UV‐Emitting Materials and Technology of Ministry of Education School of Physics Northeast Normal University 5268 Renmin Street Changchun 130024 China; ^2^ Key Laboratory of Physics and Technology for Advanced Batteries (Ministry of Education) College of Physics Jilin University Changchun 130012 China; ^3^ Graduate School of Engineering Toin University of Yokohama 1614 Kurogane‐cho, Aoba Yokohama Kanagawa 225–8503 Japan

**Keywords:** Cs_2_AgBiBr_6_, DFT calculations, double perovskite solar cells, M_2_X‐Type MXenes, strategic screening

## Abstract

MXenes have demonstrated exceptional performance in energy applications, yet their potential in photovoltaic systems, particularly in lead‐free perovskite solar cells (PSCs), remains underexplored, with no strategic studies addressing how MXene composition influences critical photovoltaic performance. Here, we present the first strategic screening of M_2_X‐type MXenes (including Hf_2_CT_x_, Zr_2_CT_x_, Ta_2_CT_x_, Nb_2_CT_x_, Mo_2_CT_x_, and V_2_CT_x_) for rational heterojunction design with Cs_2_AgBiBr_6_, focusing on the interplay between properties of MXenes and photovoltaic performance. Density functional theory (DFT) calculations reveal that MXenes can induce substantial electronic states near the Fermi level, creating superior charge transfer paths in Cs_2_AgBiBr_6_, with V_2_CT_x_ exhibiting the lowest interfacial contact barrier and the highest carrier transfer efficiency. Complementary ab initio molecular dynamics (AIMD) simulations coupled with X‐ray photoelectron spectroscopy analyses further demonstrate that the functional termination groups like ─F in MXenes effectively passivate Br vacancies in Cs_2_AgBiBr_6_, thereby enhancing crystallization and suppressing defect densities. Consequently, the experimental results yielded a trend in line with the calculations, and the power conversion efficiency (PCE) of the device with V_2_CT_x_ represented a 36% improvement, accompanied by exceptional stability. By establishing quantitative structure‐property relationships between MXenes and Cs_2_AgBiBr_6_, this work provides a universal materials selection paradigm for developing high‐performance, environmentally‐friendly photovoltaic technologies.

## Graphical abstract

The first strategic screening of M2X‐type MXenes for heterojunction design with Cs2AgBiBr6 is investigated by theory and experimental validation, revealing the role of transition metal layers, surface terminations, and work functions in enhanced photovoltaic performances. This work provides a universal materials selection paradigm for developing high‐performance, environmentally friendly photovoltaic technologies.

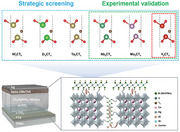

## 1 Introduction

MXenes, a large family of two‐dimensional (2D) transition metal carbides and nitrides, have garnered significant attention for their exceptional properties and versatile applications.^[^ ^1^, ^2^, ^3^ ^]^ Owing to their unique properties of high electrical conductivity, tunable surface terminations, tunable work functions, mechanical flexibility, and hydrophilicity,^[^ ^4^, ^5^, ^6^ ^]^ MXenes have demonstrated remarkable performance in energy storage,^[^ ^7^ ^]^ catalysis,^[^ ^8^ ^]^ sensors^[^ ^9^ ^]^ and electromagnetic interference shielding since their discovery in 2011.^[^ ^10^ ^]^ The typical formula of MXenes is M_n+1_X_n_T_x_, where M denotes an early transition metal, X indicates carbon and/or nitrogen, and T_x_ represents the surface terminations (such as ═O, ─OH, ─F).^[^ ^11^ ^]^ This compositional variety, combined with their rich surface termination groups, enables MXenes diversity in properties and capability of precise tailoring.^[^ ^12^ ^]^ Thus, the strategic screening of MXenes is crucial for their rapid, accurate, and specific applications.^[^ ^13^ ^]^ For instance, in lithium‐sulfur batteries, Liu et al. developed a model to accurately predict the thermodynamic energy barrier of the rate‐limiting step in Li−S batteries that related the local chemical reactivity of the MXene sites to the p‐band center of the terminations and the electronegativity of subsurface transition metals. Thus they screened a large library of MXenes (27 types of MXenes) and rapidly identified specific materials as potential cathodic catalysts.^[^ ^14^ ^]^ In catalysis, their tunable terminations and transition metal layers have been screened and leveraged to enhance hydrogen evolution reactions (HER) by Li et al, which further paved the way for designing excellent HER catalyst candidates.^[^ ^15^ ^]^ However, despite their success in these fields, there is no strategic screening of MXenes in perovskite solar cells (PSCs), particularly in environmental‐friendly lead‐free PSCs, addressing how MXene composition influences critical photovoltaic parameters such as carrier transport, defect passivation, and interfacial energy alignment.

Recently, lead organic‐inorganic halide PSCs have been rapidly developed and achieved a certified power conversion efficiency (PCE) of 27.0%, demonstrating photovoltaic performances comparable to traditional silicon‐based solar cells.^[^ ^16^, ^17^ ^]^ However, the toxicity and the leakage risk of lead raise concerns regarding both environmental pollution and human health, along with the intrinsic instability caused by the organic components become critical issues for future commercialization.^[^ ^18^, ^19^ ^]^ To address these issues, considerable progress has been made in the exploration of efficient inorganic lead‐free alternatives as the absorber layer, and Cs_2_AgBiBr_6_ double perovskite material has been demonstrated as a promising candidate.^[^ ^20^ ^]^ This double perovskite structure not only avoids the use of lead but also exhibits unique advantages such as high absorption coefficient, tunable bandgap, long carrier recombination life, and excellent thermal and optical stability, which are essential for improving the reliability of PSCs and facilitating the commercialization progress.^[^ ^21^, ^22^ ^]^ Despite these advantages, Cs_2_AgBiBr_6_ PSCs still suffer from intrinsic limitations, including a wide indirect bandgap, poor carrier mobility, and suboptimal crystallization, which result in significant energy losses and restrict PCEs to those of lead‐based devices.^[^ ^18^ ^]^

To address these challenges, numerous attempts have been explored, including additive engineering, antisolvent treatment, interface engineering, low‐pressure assisted solution processing, etc., laying the foundation for rapid development.^[^ ^23^, ^24^ ^]^ Tang et al. explored alkali metal ions as mediators to regulate the crystal lattice and quality of Cs_2_AgBiBr_6_, achieving a PCE of 2.57%.^[^ ^25^ ^]^ Wang et al. extended the absorption edge to 750 nm by constructing dye/Cs_2_AgBiBr_6_ heterojunctions, yielding a PCE of 4.23%.^[^ ^26^ ^]^ To further narrow the bandgap, Sui et al utilized a hydrogenation method that modified the bandgap of Cs_2_AgBiBr_6_ films from 2.18 to 1.64 eV, accomplishing more efficient absorption of visible light.^[^ ^27^ ^]^ Additionally, the mismatched band alignments at the Cs_2_AgBiBr_6_ and transport layers were explored by Sun et al. by introducing an effective TPTI‐TPA2F hole transport material with a deep level.^[^ ^28^ ^]^ Among these, poor crystallization and suboptimal carrier transport pathways of Cs_2_AgBiBr_6_ often result in significant energy losses, limiting the overall efficiency of the device.^[^ ^29^, ^30^ ^]^ Therefore, a deeper understanding and effective methods for better crystallization, defects passivation, and efficient carrier transfer of Cs_2_AgBiBr_6_ are essential for driving further advancements. In our previous work, Ti_3_C_2_T_x_ MXene was explored as an additive in Cs_2_AgBiBr_6_ layer.^[^ ^31^ ^]^ The introduction of Ti_3_C_2_T_x_ effectively optimized the crystallization of Cs_2_AgBiBr_6_ layer, giving rise to facilitated carrier transfer, reduced defect density, and less recombination, and the photovoltaic performance and long‐term stability were thus improved. Then we also forecasted that introducing MXenes with higher work function than Ti_3_C_2_T_x_ can reach better modulations through the simple Schottky‐Mott model. Besides, it has been demonstrated that MXenes with tunable work functions optimize band alignment and enhance carrier extraction, while the specific terminations might passivate defects and improve crystallization in lead‐based PSCs.^[^ ^32^, ^33^ ^]^ However, existing studies have been limited to isolated MXene (mainly Ti_3_C_2_T_x_), no strategic study has explored the relationship between MXene chemistry (including transition metal layers, surface terminations, and work functions) and photovoltaic performance in PSCs, nor has any work established general design principles for MXene‐perovskite heterojunctions. Given the vast compositional diversity of MXenes and their potential to address multiple challenges in Cs_2_AgBiBr_6_ PSCs simultaneously, it is worth investigating and screening the further regulations of Cs_2_AgBiBr_6_ by different MXenes through theory and experiments, thus realizing performance improvement and long‐term stability for future commercialization.

In this work, we present the first comprehensive investigation of M_2_X‐type MXenes (including Hf_2_CT_x_, Zr_2_CT_x_, Ta_2_CT_x_, Nb_2_CT_x_, Mo_2_CT_x_, and V_2_CT_x_) with high work functions for optimizing lead‐free Cs_2_AgBiBr_6_ PSCs. DFT calculations revealed that all investigated MXenes could enable substantial electronic states presented at the Fermi level in heterojunction, indicating MXenes can serve as superior charge transfer paths to facilitate carrier transfer in Cs_2_AgBiBr_6_ films. Notably, V_2_CT_x_ MXene demonstrated the lowest contact barrier of −0.375 eV and suggests the highest interfacial electron transfer value. Furthermore, ab initio molecular dynamics (AIMD) simulations, corroborated by experimental evidence, clarified the effective passivation on the Br vacancies in Cs_2_AgBiBr_6_ films by MXenes. This passivation mechanism renders better crystallization and suppresses the loss of photovoltaic performance due to defects. Consequently, the experimental results yielded a trend in line with the calculations, and the PCE of the device with V_2_CT_x_ MXene regulation represented a 36% improvement over the pristine device, while simultaneously achieving enhanced long‐term stability that over 80% of initial PCE after 1200 hours under ambient conditions.

## 2 Results and Discussion

### 2.1 Strategic Screening of M_2_X‐type MXenes for Cs_2_AgBiBr_6_

According to our previous work, we focused on the (100) plane exposed by the most stable CsBr of Cs_2_AgBiBr_6_, denoted as Cs_2_AgBiBr_6_ (100) in the following discussion.^[^ ^31^ ^]^ The band structure and the partial density of states of Cs_2_AgBiBr_6_ were obtained as shown in Figure S1 and its intrinsic properties were summarized in Table S1. The Cs_2_AgBiBr_6_ (100) is an indirect band gap semiconductor with a bandgap of 1.151 eV along with an ionization potential of 5.636 eV and an electron affinity of 4.485 eV. This bandgap is a little smaller than the experimental results owing to the underestimation of the bandgap in the calculations by Perdew–Burke–Ernzerhof (PBE) functional, in which the excited state of the structure is not considered when solving the K–S equation.^[^ ^34^ ^]^ Despite the absolute bias, the accuracy of the PBE functional is convincing in evaluating the relative value of the band edge.^[^ ^35^, ^36^ ^]^ The layer spacing of the heterojunction models before and after geometric optimization is summarized in Table S2. Then, the crystal structures and intrinsic properties of Hf_2_CT_x_, Zr_2_CT_x_, Ta_2_CT_x_, Nb_2_CT_x_, Mo_2_CT_x_, and V_2_CT_x_ with full ‐O groups are further shown in Figure S2 and in Table S3. According to Schottky‐Mott theory, these MXenes with high work functions tend to form the p‐type contact with Cs_2_AgBiBr_6_, in which the contact barriers can be calculated to be 0.333, 0.309, 0.201, −0.067, −0.712, −0.990 eV in sequence according to the Schottky–Mott formula. Thus, all six kinds of MXenes have low hole barriers, where Nb‐based, Mo‐based, and V‐based MXenes form p‐type ohmic contacts with Cs_2_AgBiBr_6_, which are effective for interface carrier transport. It is important to note that this is only a simple prediction, and the actual Schottky barrier needs to be further calculated in the MXenes/Cs_2_AgBiBr_6_ heterojunctions.

**Figure 1:**
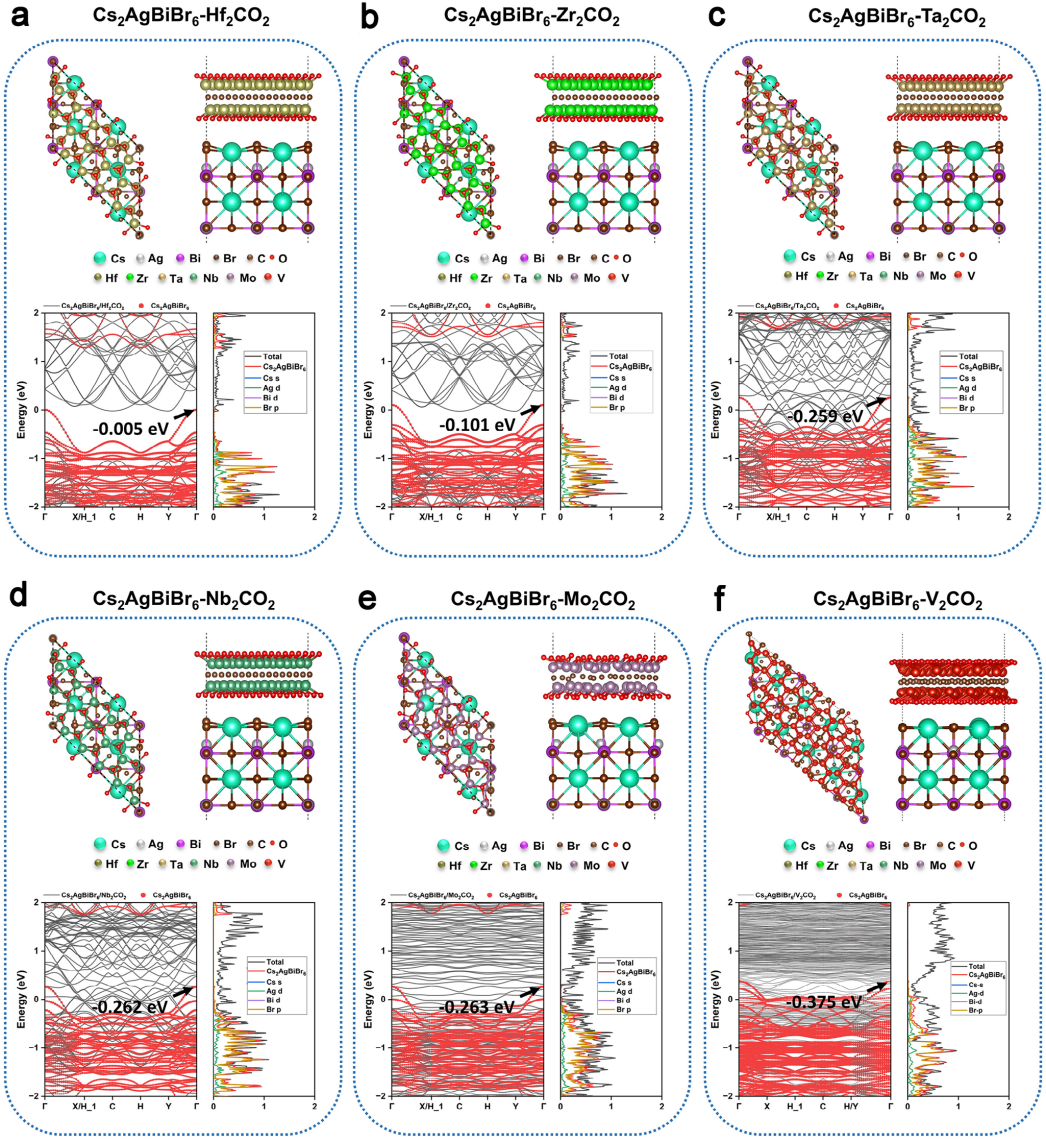
Electronic properties of heterojunctions. Crystal structure diagrams, the projected band structures and density of states for (a) Cs_2_AgBiBr_6_‐Hf_2_CO_2_, (b) Cs_2_AgBiBr_6_‐Zr_2_CO_2_, (c) Cs_2_AgBiBr_6_‐Ta_2_CO_2_, (d) Cs_2_AgBiBr_6_‐Nb_2_CO_2_, (e) Cs_2_AgBiBr_6_‐Mo_2_CO_2_, (f) Cs_2_AgBiBr_6_‐V_2_CO_2_ heterojunctions.

To minimize lattice mismatch, we rotated and extended the unit cells of Cs_2_AgBiBr_6_ and MXenes. The final models of Cs_2_AgBiBr_6_‐Hf_2_CO_2_, Cs_2_AgBiBr_6_‐Zr_2_CO_2_, Cs_2_AgBiBr_6_‐Ta_2_CO_2_, Cs_2_AgBiBr_6_‐Nb_2_CO_2_, Cs_2_AgBiBr_6_‐Mo_2_CO_2_ and Cs_2_AgBiBr_6_‐V_2_CO_2_ heterojunctions contains 115, 115, 115, 115, 115 and 250 atoms respectively with maximum lattice mismatch of 4.1%. After structural relaxation, the projected band structure and density of state for these heterojunctions are further shown in **Figure** **1**, in which all the six MXenes could form ohmic contacts with Cs_2_AgBiBr_6_ as the VBM of Cs_2_AgBiBr_6_ in heterojunctions pass through the Fermi level and move upward by 0.005, 0.101, 0.259, 0.262, 0.263, and 0.375 eV, respectively (Table S4). The density of states shows that there exist abundant electronic states in all six heterojunctions at the Fermi level compared with the original absence of any electronic states, suggesting that MXenes can indeed act as conductive additives to promote carrier transport in Cs_2_AgBiBr_6_. Among them, Cs_2_AgBiBr_6_‐V_2_CO_2_ possesses the smallest Schottky barrier, which suggests it would be the most suitable additive with the best performance among those screened. In addition, Cs_2_AgBiBr_6_ also have considerable bandgaps along with the absence of interstitial states, which show its intrinsic semiconductor characteristic is preserved and is conducive to the realization of optoelectronic properties.

**Figure 2:**
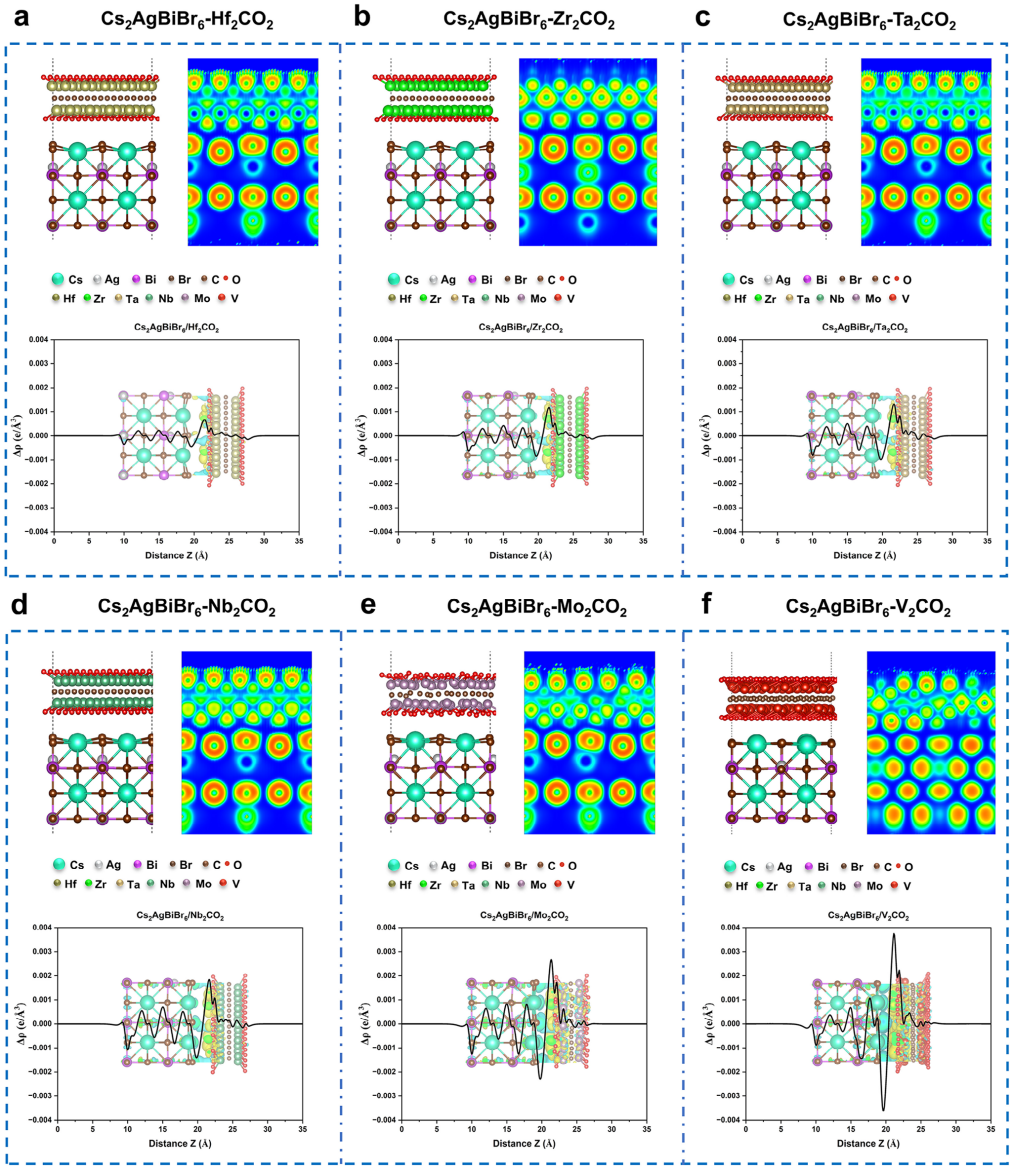
Interfacial properties between Cs_2_AgBiBr_6_ and different M_2_X‐type MXenes. Crystal structure, electron localization function and difference charge density (Isosurface level is determined at the same level.) of (a) Cs_2_AgBiBr_6_‐Hf_2_CO_2_, (b) Cs_2_AgBiBr_6_‐Zr_2_CO_2_, (c) Cs_2_AgBiBr_6_‐Ta_2_CO_2_, (d) Cs_2_AgBiBr_6_‐Nb_2_CO_2_, (e) Cs_2_AgBiBr_6_‐Mo_2_CO_2_, (f) Cs_2_AgBiBr_6_‐V_2_CO_2_.

To explore the interfacial properties between Cs_2_AgBiBr_6_ and different M_2_X‐type MXenes, the electronic localization functions (ELFs) and differential charge densities of six heterojunction systems are shown in **Figure** **2**. The ELFs indicate the absence of free electronic states at the interfaces due to the passivation of the MXene surfaces by oxygen terminal groups. These groups form a shielding layer, reducing Fermi‐level pinning effects during contact formation, and thus facilitating the effective regulation of the Cs_2_AgBiBr_6_ band alignment via different work functions of MXenes, ultimately enabling the formation of Ohmic contacts. This observation also explains why the intrinsic semiconductor characteristic of Cs_2_AgBiBr_6_ can be reserved. The difference charge density analyses reveal the consistent electron transfer from the Cs site in the perovskite to the O surface in MXene for all six heterojunction systems. Among these, the V‐based MXene system exhibits the largest interfacial charge transfer, correlating with its lowest contact barrier. The interfacial charge transfer magnitude follows the order Hf_2_CT_x_ < Zr_2_CT_x_ < Ta_2_CT_x_ < Nb_2_CT_x_ < Mo_2_CT_x_ < V_2_CT_x_, which aligns with the trend in MXene work functions (Hf_2_CT_x_ < Zr_2_CT_x_ < Ta_2_CT_x_ < Nb_2_CT_x_ < Mo_2_CT_x_ < V_2_CT_x_) and Schottky barriers (Hf_2_CT_x_ > Zr_2_CT_x_ > Ta_2_CT_x_ > Nb_2_CT_x_ > Mo_2_CT_x_ > V_2_CT_x_). These findings further highlight the potential of MXenes in regulating the band alignment of Cs_2_AgBiBr_6_. Simultaneously, the passivation of the MXene surface by oxygen groups mitigates interfacial interactions, preserving the intrinsic properties of the double perovskite, which is crucial for its optoelectronic applications and offers a promising route to enhance device performance. The results underscore the potential to predict contact barriers between MXenes and Cs_2_AgBiBr_6_, enabling a priori assessment of their impact on perovskite performance.

Besides, to further explore the influence of other surface groups on the charge distribution, we summarized the layer spacing of the heterojunction models with mixed functional groups before and after geometric optimization in Table S5 and established heterojunction models containing mixed groups (O, OH, F): Cs_2_AgBiBr_6_‐Hf_2_CO_1.534_F_0.333_(OH)_0.133_, Cs_2_AgBiBr_6_‐Zr_2_CO_1.534_F_0.333_(OH)_0.133_, Cs_2_AgBiBr_6_‐Ta_2_CO_1.534_F_0.333_(OH)_0.133_, Cs_2_AgBiBr_6_‐Nb_2_CO_1.534_F_0.333_(OH)_0.133_, Cs_2_AgBiBr_6_‐Mo_2_CO_1.534_F_0.333_(OH)_0.133_, Cs_2_AgBiBr_6_‐V_2_CO_1.588_F_0.294_(OH)_0.118_. Here, oxygen groups account for ≈75% of the proportion because of the leading role in the synthesis process, while the F group and OH group occupy 25%. After geometric optimization, their ELFs reflect in the bonding situation at the interface and differential charge density that evaluate the distribution of interface charges when forming contact for heterojunction systems of mixed groups, as shown in Figure S3. Even after considering the mixed groups, the ELFs reveal that there was still no strong bonding at the interface between Cs_2_AgBiBr_6_ and MXenes, which indicates that the surface groups of MXenes have the same effect, that is, to passivate the surface of MXenes and reserve intrinsic semiconductor characteristic of Cs_2_AgBiBr_6_. The charge density difference was calculated using the following formula to evaluate the distribution of interface charges when forming contact:

(1) $$\Delta \rho =\rho_{heter}-\rho_{MXene}-\rho_{per}$$

where ρ_heter_, ρ_MXene_, and ρ_per_ denote the charge densities of Cs_2_AgBiBr_6_/MXene, MXene, and Cs_2_AgBiBr_6_ perovskite. The difference in charge density still shows the electron transfer from the Cs site in the perovskite to the surface in MXene for all six heterojunction systems containing mixed groups. Among these, the V‐based MXene system still exhibits the largest interfacial charge transfer, which is consistent with the situation of pure oxygen groups. In fact, even for the V‐based heterojunction, the peak value of interface charge transfer is also <0.004 e Å^−^^3^, this weak redistribution of interfacial charges does not affect the carrier transport during the subsequent process.

### 2.2 Characterization of Crystal Quality and Electrical Properties of Heterojunctions

Therefore, we experimentally chose and prepared the mono‐layer M_2_X‐type MXenes of Nb_2_CT_x_, Mo_2_CT_x_, and V_2_CT_x_ by exfoliating the A layers from corresponding MAX phases (Nb_2_AlC, Mo_2_Ga_2_C, and V_2_AlC), the specific preparation methods are described in the experimental section. Figure S4 exhibits the X‐ray diffraction (XRD) patterns of these three MXenes before and after exfoliating from the original MAX phases and confirms the structural changes. After etching processes, the most characteristic (103) peaks of the Nb_2_AlC MAX phase at 38.8° (2θ), Mo_2_AlC MAX phase at 37.6° (2*θ*), V_2_AlC MAX phase at 41.3° (2*θ*) are all disappeared. The corresponding (002) diffraction peak shift from 12.9° to 7.1°, 10.0° to 7.2° and 13.6° to 7.7° in sequence, demonstrating the successful exfoliation of Nb_2_CT_x_, Mo_2_CT_x_ and V_2_CT_x_.^[^ ^37^, ^38^, ^39^ ^]^ Besides, transmission electron microscopy (TEM) images of these three MXenes’ nanosheets are measured and shown in Figure S5, in which mono‐layered Nb_2_CT_x_, Mo_2_CT_x_, and V_2_CT_x_ can be clearly recognized and further indicate the successful fabrication. After that, heterojunction Cs_2_AgBiBr_6_ of these M_2_X‐type MXenes were constructed.

**Figure 3:**
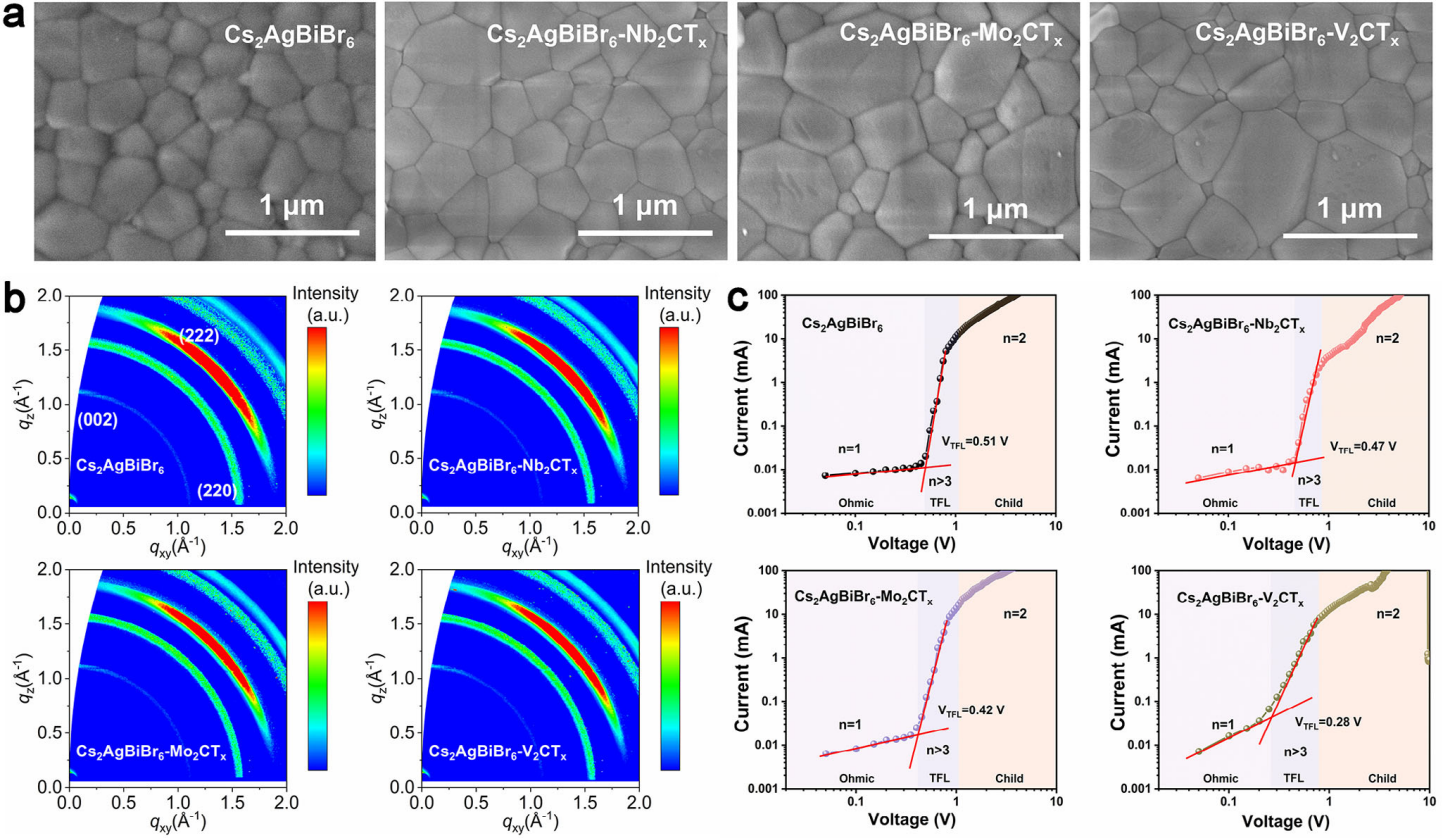
Crystal quality and electrical properties of Cs_2_AgBiBr_6_ films with different M_2_X‐type MXenes. (a) SEM images and (b) GIWAXS patterns of pristine Cs_2_AgBiBr_6_ films fabricated by spin‐coating and those modified with Nb_2_CT_x_, Mo_2_CT_x_, and V_2_CT_x_. (c) SCLC curves for the device based on pristine Cs_2_AgBiBr_6_ and those modified with Nb_2_CT_x_, Mo_2_CT_x_, and V_2_CT_x_.

By clarifying the effects of MXenes on carrier transport of the heterojunctions, we experimentally explored the crystal quality and electrical properties of Cs_2_AgBiBr_6_ films with different M_2_X‐type MXenes. The XRD patterns of pristine Cs_2_AgBiBr_6_ and Cs_2_AgBiBr_6_ with different MXenes modifications were studied as shown in Figure S6a. All the films exhibit similar diffraction peaks referred to Cs_2_AgBiBr_6_ and there are no other peaks of intermediate phases appearing, indicating the pure phases for all films.^[^ ^40^ ^]^ Additionally, the full‐width half‐maximum (FWHM) at around 32.2° corresponding to (400) peaks follow the order of Cs_2_AgBiBr_6_ > Cs_2_AgBiBr_6_‐Nb_2_CT_x_ > Cs_2_AgBiBr_6_‐Mo_2_CT_x_ > Cs_2_AgBiBr_6_‐V_2_CT_x_ (Figure S6b). The narrow FWHM suggests improved crystallization of Cs_2_AgBiBr_6_ by MXenes modification, in which V_2_CT_x_ exhibits the best optimization performance. Then, the scanning electron microscope (SEM) images of these four films were obtained as shown in **Figure** **3**a. All these films are dense, and uniform without pin‐holes, and the grain sizes are increased after MXenes modification, in which the Cs_2_AgBiBr_6_‐V_2_CT_x_ exhibits the largest grain size. The grain size distribution of Cs_2_AgBiBr_6_ films with or without MXenes modification are analyzed by linear intercept method and shown in Figure S7. The average grain sizes are calculated to be 364, 425, 498, and 516 nm for Cs_2_AgBiBr_6_, Cs_2_AgBiBr_6_‐Nb_2_CT_x_, Cs_2_AgBiBr_6_‐Mo_2_CT_x_, and Cs_2_AgBiBr_6_‐V_2_CT_x_, respectively, which are in consistent with the XRD results above. As for the best performance of Cs_2_AgBiBr_6_‐V_2_CT_x_, the SEM images for those films with different V_2_CT_x_ mixing concentrations were obtained in the meantime (Figure S8). Among them, a V_2_CT_x_ mixing concentration of 0.02 mg mL^−1^ results in the optimal crystallization, thus the concentration indicated subsequently of Cs_2_AgBiBr_6_‐V_2_CT_x_ are all 0.02 mg mL^−1^. Owing to the improved crystallization, UV–vis absorption peak at 438 nm of Cs_2_AgBiBr_6_ are also increased after MXenes modification (Figure S9), and the most ascended absorption was obtained for Cs_2_AgBiBr_6_‐V_2_CT_x_, indicating optimized optical properties. Besides, UV photoelectron spectroscopy (UPS) measurements on the pristine Cs_2_AgBiBr_6_ film and that modified by the representative V_2_CT_x_ MXene are performed to verify the consistency of the calculated results. As presented in Figure S10, the Fermi level and VB of Cs_2_AgBiBr_6_ are determined to be −5.077 and −6.226 eV, thus the CB can be calculated to be −3.646 eV according to the UV–vis absorption measurements (Figure S9). After introducing V_2_CT_x_ MXene, the Fermi level of heterojunction exhibits a downward trend and is determined to be 5.255 eV due to the high work function of V_2_CT_x_, which suggests promoted suitability for carrier transport.^[^ ^41^ ^]^

To further investigate the crystallization kinetics that may affect the facet orientation arrangement of Cs_2_AgBiBr_6_ double perovskite,^[^ ^42^ ^]^ the synchrotron‐radiation‐based grazing‐incidence wide‐angle X‐ray scattering (GIWAXS) were performed on the surfaces of Cs_2_AgBiBr_6_ films with or without MXenes modification as shown in Figure 3b. The patterns of Cs_2_AgBiBr_6_ films with MXenes‐modified exhibit more favorable (002) diffraction peaks aggregated scattering ring at *q*_xy_ ≈ 1.12 Å, indicating better facet orientation of Cs_2_AgBiBr_6_ films. More (002) facets are beneficially aligned parallel to the substrate, thus rendering high‐quality oriented perovskite films.^[^ ^43^ ^]^ Moreover, space charge‐limited current (SCLC) measurements of the devices based on Cs_2_AgBiBr_6_, Cs_2_AgBiBr_6_‐Nb_2_CT_x_, Cs_2_AgBiBr_6_‐Mo_2_CT_x_, and Cs_2_AgBiBr_6_‐V_2_CT_x_ are displayed in Figure 3c. The three regions are distinguished as *n* = 1 region with an ohmic contact, *n* = 2 region a Child's law, and a trap‐filled limit (*n* > 3). The trap state density (*N*_trap_) of the Cs_2_AgBiBr_6_ perovskite film can be obtained according to the following equation:^[^ ^44^ ^]^

(2) $$V_{TFL}=\frac{eN_{trap}L^{2}}{2\varepsilon \varepsilon_{0}}\;$$

where *V_TFL_* represents the onset voltage of the trap‐filled limit (TFL) region, *e* stands for the charge of one electron, L refers to the thickness of Cs_2_AgBiBr_6_ films, ε represents the relative dielectric constant of Cs_2_AgBiBr_6_ and ε_0_ is the vacuum permittivity. The *V_TFL_* of the Cs_2_AgBiBr_6_, Cs_2_AgBiBr_6_‐Nb_2_CT_x_, Cs_2_AgBiBr_6_‐Mo_2_CT_x_, and Cs_2_AgBiBr_6_‐V_2_CT_x_ are 0.51, 0.47, 0.42 and 0.28 V, thus the *N_trap_* is calculated to be 2.51 × 10^15^, 2.31 × 10^15^, 2.06 × 10^15^, and 1.38 × 10^15^ cm^−3^, respectively, demonstrating the suppressed trap densities of Cs_2_AgBiBr_6_ films with MXenes modification and the best performance induced by V_2_CT_x_. In addition, the stress‐based analyses are crucial for demonstrating the crystallinity of perovskite after mixing with MXenes.^[^ ^45^ ^]^ Thus, we explored the films of Cs_2_AgBiBr_6_ and Cs_2_AgBiBr_6_‐V_2_CT_x_ through grazing incident X‐ray diffraction (GIXRD) as presented in Figure S11. By varying φ (the grazing incidence angle) from 0.2° to 1.0°, the penetration depth of X‐ray into Cs_2_AgBiBr_6_ films gets deeper, and the highest (400) peak shifts toward a lower angle. According to the Bragg equation of *nλ* = 2*d*sinθ, this shift indicates the expanded interplanar crystal space of Cs_2_AgBiBr_6_ due to the residual strain. After introducing V_2_CT_x_, the shift of (400) peak becomes slighter, indicating the suppression of residual strains and well‐matched with the improved crystallinity of perovskite films as shown above.

### 2.3 Passivation on Cs_2_AgBiBr_6_ Films by MXenes

**Figure 4:**
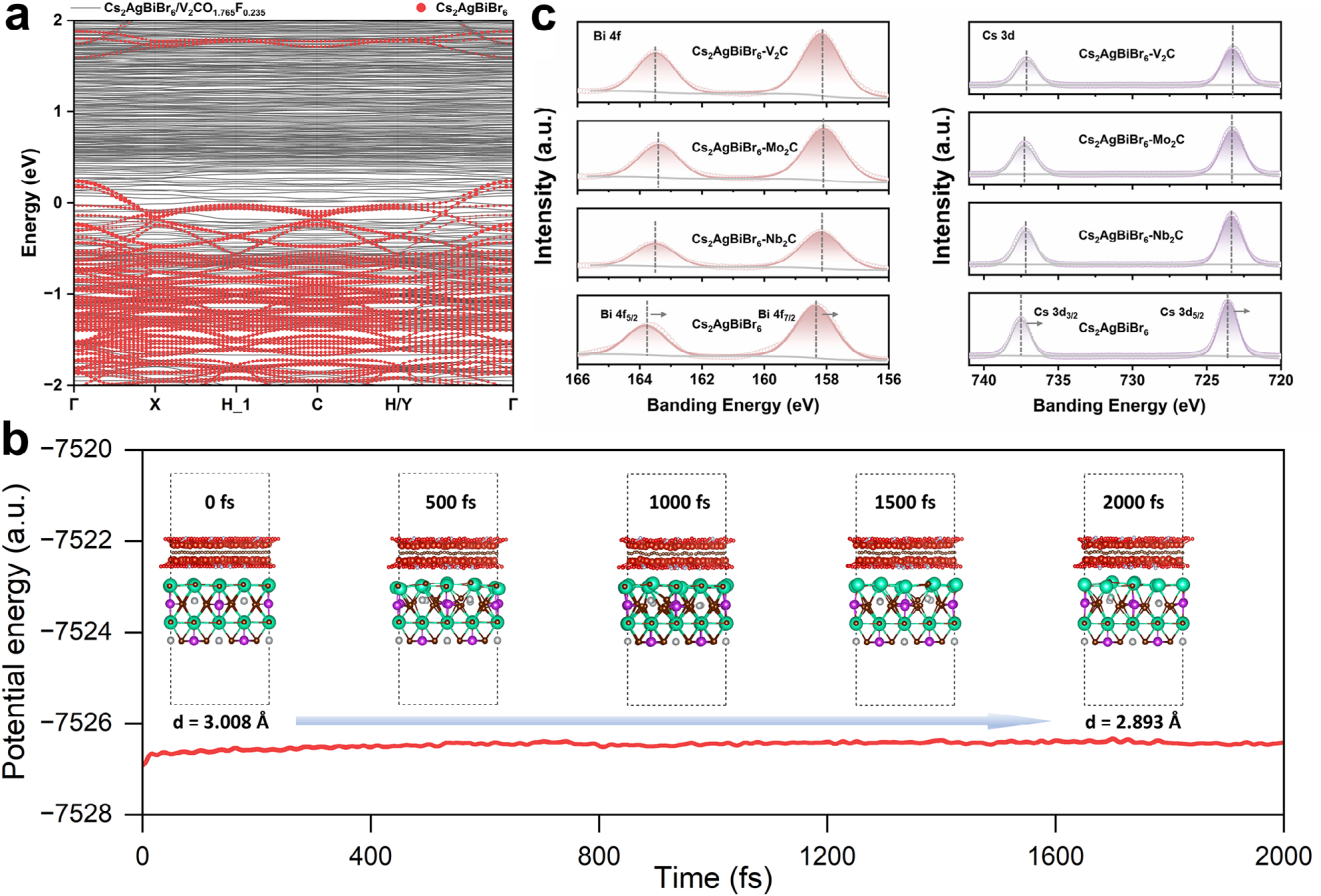
Passivation on Cs_2_AgBiBr_6_ films by MXenes. (a) Band structure calculations of Cs_2_AgBiBr_6_ with V_2_CO_1.765_F_0.235_. (b) Interfacial evolution constructed by AIMD simulation of Cs_2_AgBiBr_5.875_ and V_2_CO_1.765_F_0.235_. (c) The fitted Bi 4f and Cs 3d core‐level spectra of Cs_2_AgBiBr_6_, Cs_2_AgBiBr_6_‐Nb_2_CT_x_, Cs_2_AgBiBr_6_‐Mo_2_CT_x_, Cs_2_AgBiBr_6_‐V_2_CT_x_.

In the above calculation, we only investigated the effect of MXene with full ‐O groups on Cs_2_AgBiBr_6_, but the real MXene often contains ─F groups. Therefore, we further consider the model of Cs_2_AgBiBr_6_/V_2_CO_1.765_F_0.235_ with mixed ─O/─F groups. The band structure in **Figure** **4**a shows, that after adding the ─F groups, the VBM of Cs_2_AgBiBr_6_ moves down, causing the Schottky barrier to increase from −0.375 to −0.241 eV. The nature of its ohmic contact is still preserved. Further, we constructed the heterojunction model containing 2% Br defects, noted as Cs_2_AgBiBr_5.875_/ V_2_CO_1.765_F_0.235_ and then observed the dynamic evolution process of the interfaces by AIMD simulation as shown in Figure 4b. In the 2000 fs simulation, the unsaturated coordination caused by Br defects causes the interface Cs atoms to tend toward the ─O/─F groups of MXenes along with the decreasing interface layer spacing from the original 3.008 to 2.893 Å, which reveals that MXenes can effectively passivate the vacancies of Br in Cs_2_AgBiBr_6_ films and achieve better crystallinity, thus suppressing the loss of photovoltaic performance due to defects.

The X‐ray photoelectron spectroscopy (XPS) measurements are conducted to analyze the surface chemical states of pristine Cs_2_AgBiBr_6_ and Cs_2_AgBiBr_6_ with different MXenes modifications as shown in Figure S12. The relevant peaks of Cs, Ag, Bi, Br are clearly detected, while the fitted Nb 3d core‐level spectra of Cs_2_AgBiBr_6_‐Nb_2_CT_x_, fitted Mo 3d core‐level spectra of Cs_2_AgBiBr_6_‐Mo_2_CT_x_ and fitted V 2p core‐level spectra of Cs_2_AgBiBr_6_‐V_2_CT_x_ are found (Figure S13). These results indicate the formation of Cs_2_AgBiBr_6_ double perovskite films and the successful introduction of individual MXenes. Moreover, fitted Bi 4f and Cs 3d core‐level spectra of Cs_2_AgBiBr_6_, Cs_2_AgBiBr_6_‐Nb_2_CT_x_, Cs_2_AgBiBr_6_‐Mo_2_CT_x_, Cs_2_AgBiBr_6_‐V_2_CT_x_ are further investigate as shown in Figure 4c. It can be clearly seen the characteristic peaks of Bi 4f and Cs 3d both slightly shift toward lower energy after introducing MXenes, indicating effective passivation on Br vacancy of MXenes according to calculation results.

### 2.4 Photovoltaic Performance of Cs_2_AgBiBr_6_ Devices Modified with MXenes

**Figure 5:**
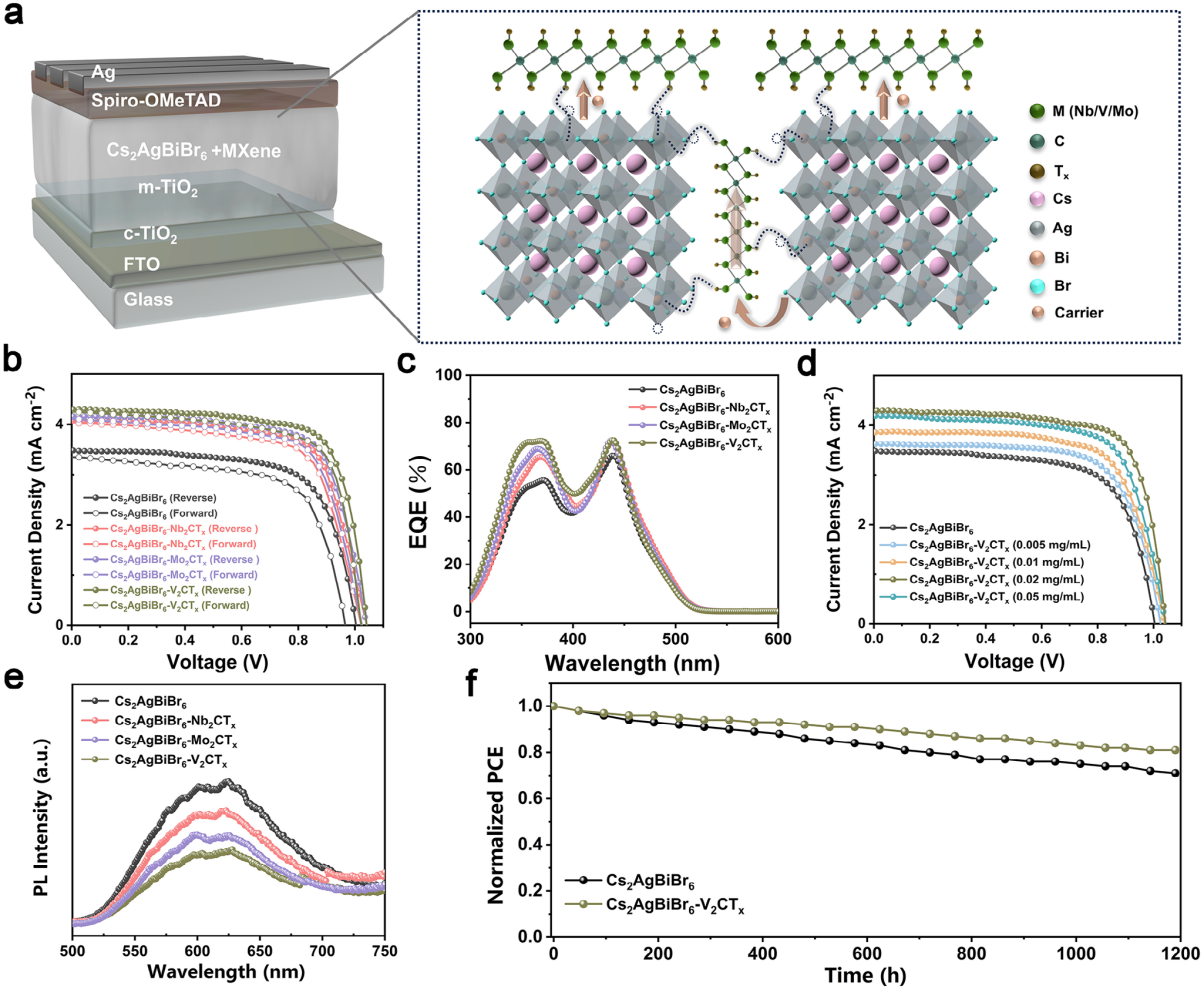
Effects of MXenes on photovoltaic performance. (a) PSC architecture of FTO/c‐TiO_2_/m‐TiO_2_/Cs_2_AgBiBr_6_‐MXene/Spiro‐OMeTAD/Ag, along with the schematic diagram of defect passivation on Cs_2_AgBiBr_6_ films by MXenes and electronic transfer paths. (b) *J–V* curves in reverse and forward scanning directions of devices based on Cs_2_AgBiBr_6_ with different MXenes modification and their (c) EQE spectra. (d) *J–V* curves of devices based on Cs_2_AgBiBr_6_‐V_2_CT_x_ with different modified concentrations. (e) Steady‐state photoluminescence spectra of Cs_2_AgBiBr_6_ films with different MXenes modification. (f) Long‐term stability measurements of unencapsulated devices in the ambient atmosphere (≈ 40 RH%) at 25 °C in the dark.

Combining the above results, the devices with an architecture of FTO/c‐TiO_2_/m‐TiO_2_/Cs_2_AgBiBr_6_‐MXene/Spiro‐OMeTAD/Ag were fabricated. The active layer of Cs_2_AgBiBr_6_ were modified with Nb_2_CT_x_, Mo_2_CT_x_, and V_2_CT_x_ in sequence, in which MXenes serve as electronic transfer paths and passivators as shown in **Figure** **5**a. The corresponding cross‐sectional SEM images of the devices are presented in Figure S14, and the Cs_2_AgBiBr_6_‐V_2_CT_x_‐based device exhibits flatter and oriented Cs_2_AgBiBr_6_ films compared with pristine Cs_2_AgBiBr_6_. The current density–voltage (*J–V*) curves with forward and reverse scans of devices based on Cs_2_AgBiBr_6_ with different MXenes modifications are presented in Figure 5b, and the relevant photovoltaic parameters are summarized in Table S6. Initially, the device based on pristine Cs_2_AgBiBr_6_ exhibits a champion PCE of 2.38% with relatively low open circuit voltage (*V_oc_*) of 1.004 V, current density (*J_sc_*) of 3.45 mAcm^−2^and fill factor (FF) of 0.691. After introducing M_2_X‐type MXenes, the parameters of the target PSCs were all got increased due to the superior carrier transfer, high crystallization of perovskite layer, and defects passivation induced by MXenes, thus rendering enhancements of PCEs and suppressed hysteresis. The hysteresis indexes (HI) are calculated to be 9.7% for Cs_2_AgBiBr_6_, 6.0% for Cs_2_AgBiBr_6_‐ Nb_2_CT_x_, 6.4% for Cs_2_AgBiBr_6_‐ Mo_2_CT_x_, 4.9% for Cs_2_AgBiBr_6_‐ V_2_CT_x_, respectively. Among them, peak performance is accomplished with the device based on Cs_2_AgBiBr_6_‐V_2_CT_x_, delivering an exceptional PCE of 3.25%. The remarkable performances are mainly attributed to its maximum V energy level difference to Cs_2_AgBiBr_6_ and minimum interfacial transfer barrier compared with Cs_2_AgBiBr_6_‐Nb_2_CT_x_ and Cs_2_AgBiBr_6_‐Mo_2_CT_x_.

Additionally, their external quantum efficiency (EQE) spectra were measured and performed in Figure 5c. The devices with MXenes modification exhibit similar response profiles but higher values from 300 to 500 nm, indicating better carrier transfer and confirming the enhancements of *J–V* results. The enhancements could be caused by the passivation of perovskite, thus we considered four models (Figure S15), the perfect Cs_2_AgBiBr_6_ perovskite, the Cs_2_AgBiBr_5.75_ perovskite with 4.1% Br vacancies, the Cs_2_AgBiBr_5.75_F_0.25_ and Cs_2_AgBiBr_5.75_Cl_0.25_ using F and Cl to replace Br vacancies, and performed band structures and density of states for these models, as show in Figure S16. The absence of Br can lead to electron enrichment in the system, causing the Fermi level to shift upward. This result indicates that Br defects and their concentration strictly control the conductive type of Cs_2_AgBiBr_6_ perovskite. If we use F and Cl from the same main group to replace Br vacancies, we find that their band structures are similar to those without defects. This is because F and Cl act as electron acceptors, causing the electrons in the system to become electrically neutral. In fact, the electron transfer from the Cs site in the perovskite to the surface in MXene has been demonstrated when forming contact from Figure 2 and Figure S3. MXenes act as electron acceptors as same as F and Cl. Therefore, introducing MXenes to perovskite can effectively compensate for the performance reduction caused by Br defects. Furthermore, our AIMD results (Figure 4c) also show that the surface F groups of MXenes tend to compensate for Br vacancies, further counteracting the effect of Br deletion. In the meantime, to further confirm the optimum compound concentration of these three MXenes, *J–V* curves of devices based on Cs_2_AgBiBr_6_‐V_2_CT_x_ (Figure 5d) with different concentrations, together with Cs_2_AgBiBr_6_‐Nb_2_CT_x_ (Figure S17) and Cs_2_AgBiBr_6_‐Mo_2_CT_x_ (Figure S18) are demonstrated. The photovoltaic performance parameters of the devices are summarized in Tables S7–S9, respectively. Similar trends can be seen in these three MXenes‐based devices that the *J_sc_*, *V_oc_*, and FF are gradually enhancing and then decreasing with increasing concentration of MXenes, and the most appropriate concentration is determined to be 0.02 mg mL^−1^.

Based on the device performances above, the photovoltaic properties are subjected to further exploration and comparation. Figure 5e exhibits the steady‐state photoluminescence (PL) analyses to evaluate the impacts of Cs_2_AgBiBr_6_ films with different MXenes and the varying concentrations of MXenes (Figure S19) on carrier transfer efficiency. The PL intensities align with the trend in Cs_2_AgBiBr_6_ > Cs_2_AgBiBr_6_‐Nb_2_CT_x_ > Cs_2_AgBiBr_6_‐Mo_2_CT_x_ > Cs_2_AgBiBr_6_‐V_2_CT_x_, and the lower PL indicates superior carrier transfer in the heterojunction. This finding is consistent with the trend of photovoltaic performances in *J–V* results. Since V_2_CT_x_ shows the best modulation properties among the three MXenes, the subsequent photovoltaic measurements are mainly focused on the comparison of Cs_2_AgBiBr_6_ and Cs_2_AgBiBr_6_‐V_2_CT_x_. Figure S20 shows steady‐state power output at the max power point (MPP) plot of the devices based on pristine Cs_2_AgBiBr_6_ and Cs_2_AgBiBr_6_‐V_2_CT_x_. Both devices present scarcely fluctuating behavior and indicate consistent power outputs, indicating the potential of stabilizing long‐term applications. Besides, the dark *J–V* curves of devices are obtained to confirm the traps of the Cs_2_AgBiBr_6_ films before and after V_2_CT_x_ modification (Figure S21). The device with V_2_CT_x_ conducts a much lower leakage current compared with that based on target Cs_2_AgBiBr_6_, indicating more electrons can effectively transfer to transport layers with less recombination.^[^ ^37^ ^]^

Moreover, curves of *J_sc_* and *V_oc_* based on Cs_2_AgBiBr_6_ and Cs_2_AgBiBr_6_‐V_2_CT_x_ with different incident light intensities are explored as performed in Figures S22 and S23. The slopes of *J_sc_* are carried out to be 0.949 and 0.982 in sequence, in which the slope of *J_sc_* versus light intensity closer to 1 demonstrates the improved carrier transfer and suppressed defects.^[^ ^46^ ^]^ The *V_oc_* versus light intensity follows the equation below:

(3) $$N=\frac{q}{k_{B}T}\;\frac{dV_{oc}}{dLnI}$$

where *N* refers to the ideal factor associated with trap‐induced non‐radiative recombination in devices. *q* stands for the charge of one electron, *k_B_* is the Boltzmann constant, *T* represents the absolute temperature and *I* is the light intensity. After fitting, the values of *N* for the devices based on Cs_2_AgBiBr_6_ and Cs_2_AgBiBr_6_‐V_2_CT_x_ are determined to be 1.69 and 1.40. This result also demonstrates a suppressed trap density and reduced recombination aroused from V_2_CT_x_. In addition, the capacitance‐voltage (Mott–Schottky) analysis of the devices based on pristine Cs_2_AgBiBr_6_ and Cs_2_AgBiBr_6_‐V_2_CT_x_ has been carried out as shown in Figure S24. *V*_bi_ values are estimated at 0.90 V for Cs_2_AgBiBr_6_‐V_2_CT_x_ and 0.88 V for Cs_2_AgBiBr_6_, which is consistent with the trend of *J–V* results. The higher *V*_bi_ suggests enhanced photogenerated carrier extraction and more effective recombination suppression. Additionally, the steeper slope in the Mott−Schottky plot for Cs_2_AgBiBr_6_‐V_2_CT_x_ indicates enhanced charge transfer and lower carrier accumulation. Furthermore, to further investigate the improved FF related to carrier transport at the interface, electrochemical impedance spectroscopy (EIS) measurements were conducted and shown in Figure S25. Fitted data from the Nyquist plots of these two devices are summarized in Table S10, in which *R_1_* represents the series resistance from the FTO substrate to external wires, and *R_2_* refers to the interfacial transport resistance. It can be seen the values of *R_1_* and *R_2_* decreased from 101.4 to 78.7 Ω and 1020.6 to 357.3 Ω, respectively. The significantly suppressed interfacial resistance demonstrates the optimized carrier transport induced by V_2_CT_x_ MXene, thus leading to higher FF and improved PCEs of the devices.

The stability of the PSCs is another important parameter for evaluating performance. Figure 5f performs the long‐term moisture stability of the unencapsulated devices based on Cs_2_AgBiBr_6_ and Cs_2_AgBiBr_6_‐V_2_CT_x_ under a relative humidity of 40% in the ambient atmosphere at 25 °C in the dark, following the ISOS‐D‐1 protocol. The device based on Cs_2_AgBiBr_6_‐V_2_CT_x_ exhibits superior stability, which underwent a loss of <20% after 1200 h of storage compared with that of 29% based on Cs_2_AgBiBr_6_. The enhanced stability is mainly attributed to the better crystallization of Cs_2_AgBiBr_6_ film with denser morphology and less defects by regulation of V_2_CT_x_ MXene. Additionally, photovoltaic parameter statistics for PSCs based on Cs_2_AgBiBr_6_, Cs_2_AgBiBr_6_‐Nb_2_CT_x_, Cs_2_AgBiBr_6_‐Mo_2_CT_x_, and Cs_2_AgBiBr_6_‐V_2_CT_x_ are summarized in Figure S26, indicating minor standards and excellent reproducibility.

## 3 Conclusion

In this work, we first demonstrate a comprehensive framework of M_2_X‐type MXenes (including Hf_2_CT_x_, Zr_2_CT_x_, Ta_2_CT_x_, Nb_2_CT_x_, Mo_2_CT_x_, and V_2_CT_x_) for rational heterojunction design with Cs_2_AgBiBr_6_. DFT calculations reveal that MXenes with high work functions induce substantial electronic states near the Fermi level and serve as efficient carrier transport pathways within the Cs_2_AgBiBr_6_ lattice. Notably, V_2_CT_x_ exhibits the lowest interfacial contact barrier and the highest carrier transfer efficiency. Complementary AIMD simulations and XPS analyses further confirm that fluorine terminations of MXenes effectively passivate Br vacancies, rendering enhanced crystallization and reduced defect density. These synergistic effects of enhanced carrier mobility, defect suppression, and optimized interfacial energy alignment collectively contribute to a 36% increase in PCE compared with the Cs_2_AgBiBr_6_‐based PSCs, accompanied by long‐term stability for applications. By integrating theoretical insights with experimental validation, this work not only demonstrates the fundamental mechanisms of interactions between MXenes and Cs_2_AgBiBr_6_ but also provides a generalizable materials design strategy for developing high‐performance, environmentally friendly optoelectronic devices, thus representing a significant step toward the practical implementation of lead‐free perovskite technologies.

## Conflict of Interest

The authors declare no conflict of interest.

## Supporting information



Supporting Information

## Data Availability

The data that support the findings of this study are available from the corresponding author upon reasonable request.
