# Supplementary material for: MXene‐Engineered Cs2AgBiBr6 Perovskite Solar Cells: Rational Screening and Interfacial Dynamics for Lead‐Free Photovoltaics
Source: Adv Sci (Weinh). 2025 Jun 29;12(33):e06567. doi: 10.1002/advs.202506567 (PMC12412566; doi:10.1002/advs.202506567)
Supplement: Supplementary file 1 — Supporting Information [file ADVS-12-e06567-s001.docx]

**Supporting Information**

MXene-Engineered Cs_2_AgBiBr_6_ Perovskite Solar Cells: Rational Screening and Interfacial Dynamics for Lead-Free Photovoltaics

Lin Yang ^a^, Tianfang Zheng ^b^, Ziyan Liu ^b^, Naoyuki Shibayama ^c^, Peng Li ^a^, Jiangang Ma ^a,^ *, Xintong Zhang ^a^, Hancheng Zhu ^a^, Xiao-Feng Wang ^b,^ *, Haiyang Xu ^a^, Yichun Liu ^a^

^a^ State Key Laboratory of Integrated Optoelectronics, Key Laboratory for UV-Emitting Materials and Technology of Ministry of Education, School of Physics, Northeast Normal University, 5268 Renmin Street, Changchun 130024, China

^b^ Key Laboratory of Physics and Technology for Advanced Batteries (Ministry of Education), College of Physics, Jilin University, Changchun 130012, China

^c^ Graduate School of Engineering, Toin University of Yokohama, 1614 Kurogane-cho, Aoba, Yokohama, Kanagawa 225-8503, Japan.

Corresponding Authors

^*^ Jiangang Ma, *E-mail*: majg@nenu.edu.cn

^*^ Xiao-Feng Wang, *E-mail*: xf_wang@jlu.edu.cn

# Experimental section

**Materials**

MAX phases (Nb_2_AlC, Mo_2_Ga_2_C, V_2_AlC) were purchased from Jilin 11 Technology Co., Ltd. HF (49 wt.%) and Ascorbic acid (C_6_H_8_O_6_ 99%) were purchased from Aladdin. Cesium bromide (CsBr), bismuth bromide (BiBr_3_), silver bromide (AgBr) and tetramethylammonium hydroxide (TMAOH 25% in water) were obtained from Alfa Aesar. Titanium (Ⅳ) isopropoxide, dimethyl sulfoxide (DMSO), *N,N*-dimethylformamide (DMF), chlorobenzene (CB), Spiro-OMeTAD and 4-tert-butylpyridine (TBP) were obtained from Sigma Aldrich. Technology Co., Ltd. TiO_2_ nanoparticles (PST-18NR) were obtained from JGC Catalysts. Chemicals Ltd. Titanium tetrachloride (TiCl_4_) was obtained from Macklin. Tert-butyl alcohol and acetonitrile were purchased from Aladdin. Lithium bis (trifluoromethane sulphonyl) imide (Li-TFSI) and were purchased from Xi’an Polymer Light Technology Corp.

**Preparation of Multi-Layered MXenes (Nb_2_CT_x_, Mo_2_CT_x_, V_2_CT_x_)**

All these three multi-layered MXenes (Nb_2_CT_x_, Mo_2_CT_x_, V_2_CT_x_) were fabricated by etching the A atomic layer (including Al and Ga) from relevant MAX phases. Specifically, 40 ml of hydrofluoric acid (HF) as etchant corresponded to 3 g of MAX powder. After the MAX phases of Nb_2_AlC and Mo_2_Ga_2_C were gradually added to HF, the mixtures were continuously stirred for 72 hours at a temperature of 55 °C. As for V_2_AlC, the temperature of stirring is 50 °C. The mixtures were then processed by multiple rounds of centrifugation deionized water at 8000 rpm until the pH value approached 7. Finally, the multi-layer MXene were dried in a vacuum at 60 °C for 12 hours.

**Fabrication of Few-Layered MXenes (Nb_2_CT_x_, Mo_2_CT_x_, V_2_CT_x_)**

Few-layer MXenes (Nb_2_CT_x_, Mo_2_CT_x_, V_2_CT_x_) were fabricated through the exfoliation of the multi-layered products mentioned above. In this process, 2 g of multi-layer MXene powder were placed in a Teflon-lined stainless-steel autoclave, along with 20 mL of deionized water, 40 mL of undiluted tetramethylammonium hydroxide (25% TMAOH), and 1.0 gram of ascorbic acid. The mixtures were delaminated at a temperature of 140 °C for 24 hours. Upon cooling to room temperature, the black products were centrifuged at 8000 rpm to separate them from any excess impurities. The centrifuged mixtures were then supplemented with the appropriate amount of deionized water and further exfoliated through sonication for 60 minutes. Finally, colloidal solutions containing few-layered MXene nanosheets were obtained by collecting the supernatant after centrifugation at 3500 rpm for 1 hour.

**Device Fabrication**

FTO glass substrates underwent sequential ultrasonic cleaning in detergent, deionized water, acetone, and ethanol (30 min each). After treatment with UV-ozone for 20 min, a compact TiO_2_ (c-TiO_2_) layer was deposited via spin-coating at4000 rpm for 30 s using 10 vol% titanium(Ⅳ) isopropoxide solution, followed by annealing at 200°C for 30 min. Mesoporous TiO_2_ (m- TiO_2_) was subsequently formed by spin-coating (2000 rpm, 30 s) an ethanol-based TiO_2_ colloidal solution (0.39 g/mL) and sintering at 500°C for 30 min. Then, the m-TiO_2_ layer was chemically treated by immersing in aqueous TiCl_4_ solution (67.5 μL TiCl_4_/10 mL H_2_O) at 70°C for 1 h, followed by secondary sintering at 500°C for 30 min. After cooling down to room temperature, perovskite precursor solution containing CsBr (1 mmol), BiBr₃ (0.5 mmol), AgBr (0.5 mmol) and M_2_X-type MXenes with different concentrations in DMSO was spin-coated (3000 rpm, 60 s) and annealed at 280°C for 30 min to crystallize the Cs_2_AgBiBr_6_ layer. Next, Spiro-OMeTAD solution (72.3 mg in chlorobenzene with 30 μL 4-tert-butylpyridine and 35.5 μL Li-TFSI/acetonitrile (260 mg/mL)) was spin-coated (4000 rpm, 30 s) onto the perovskite layer. Finally, silver electrodes were thermally evaporated through a shadow mask to complete the device fabrication.

**Characterizations**

X-ray diffraction (XRD) patterns were acquired using a Bruker D8 Advance diffractometer (CuKα radiation, λ = 1.5406 Å) at room temperature. Scanning electron microscopy (SEM) measurements were examined through a field emission scanning electron microscopy (Regulus 8100). TEM images were obtained by a JEM-2200FS (JEOL). UV-visible absorption spectra were recorded using a Shimadzu UV-1900 spectrophotometer. Steady-state photoluminescence (PL) spectra were measured with a Shimadzu RF-5301PC spectrometer using 440 nm excitation wavelength. The X-ray photoelectron spectroscopy (XPS) results were performed by ThermoFischer ESCALAB 250Xi. The 2D-GIWAXS images represented in the reciprocal lattice space were conducted at BL19B2 beamline of SPring-8. The perovskite films were irradiated with an X-ray energy of 12.39 keV at a fixed-incident angle on the order of 0.5 through a Huber diffractometer. 2D-GIWAXS images were recorded using a 2D Pilatus 300 K image detector. Current density-voltage (*J-V*) characteristics were measured under AM 1.5G illumination (100 mW/cm^2^) using a Keithley 2400 source meter. External quantum efficiency (EQE) spectra were acquired in ambient conditions using a commercial EQE setup (CrowntechQTest Station 1000AD, SOFN INSTRUMENTS CO., LTD). Electrochemical impedance spectroscopy (EIS) measurements were conducted using a VSP multi-channel potentiostat- galvanostatic system (Biologic, France).

**Computational methods**

All DFT calculations except for ab initio molecular dynamics (AIMD) simulations are perform by using the Vienna Ab Initio Simulation Package (VASP) along with the projector augmented wave (PAW) method.^[1, 2]^ And we employ the generalized gradient approximation (GGA) of the Perdew–Burke–Emzerhof (PBE) method to consider the exchange correlation effect.^[3]^ The Grimme’s D3 methods are applied to include vdW corrections for all DFT calculations.^[4, 5]^ The cut-off energy and the energy convergence criterion are respectively 500 eV, 10^-6^ eV. For structural optimization, the force convergence of all MXene/Cs_2_AgBiBr_6_ heterojunctions are 0.05 eV/Å with 1×1×1 Gamma scheme. Further, we adopted the spacing of reciprocal space sampling of 0.188 Å-1 to calculate the relevant property. The band structure and charge density difference results are analyzed by the VASPKIT package.^[6]^ All the crystal structures are visualized by using VESTA.^[7]^ For all MXenes, O and F functional groups follow the ABC arrangement. The 2000 fs NVT AIMD simulation with time step of 1 fs at 500 K for Cs_2_AgBiBr_5.875_/V_2_CO_1.765_F_0.235_ was performed using the CP2K package,^[8]^ employing PBE method, D3 vdW corrections and OT algorithms. The CUTOFF and REL_CUTOFF are set to 500 and 50 Ry, respectively.


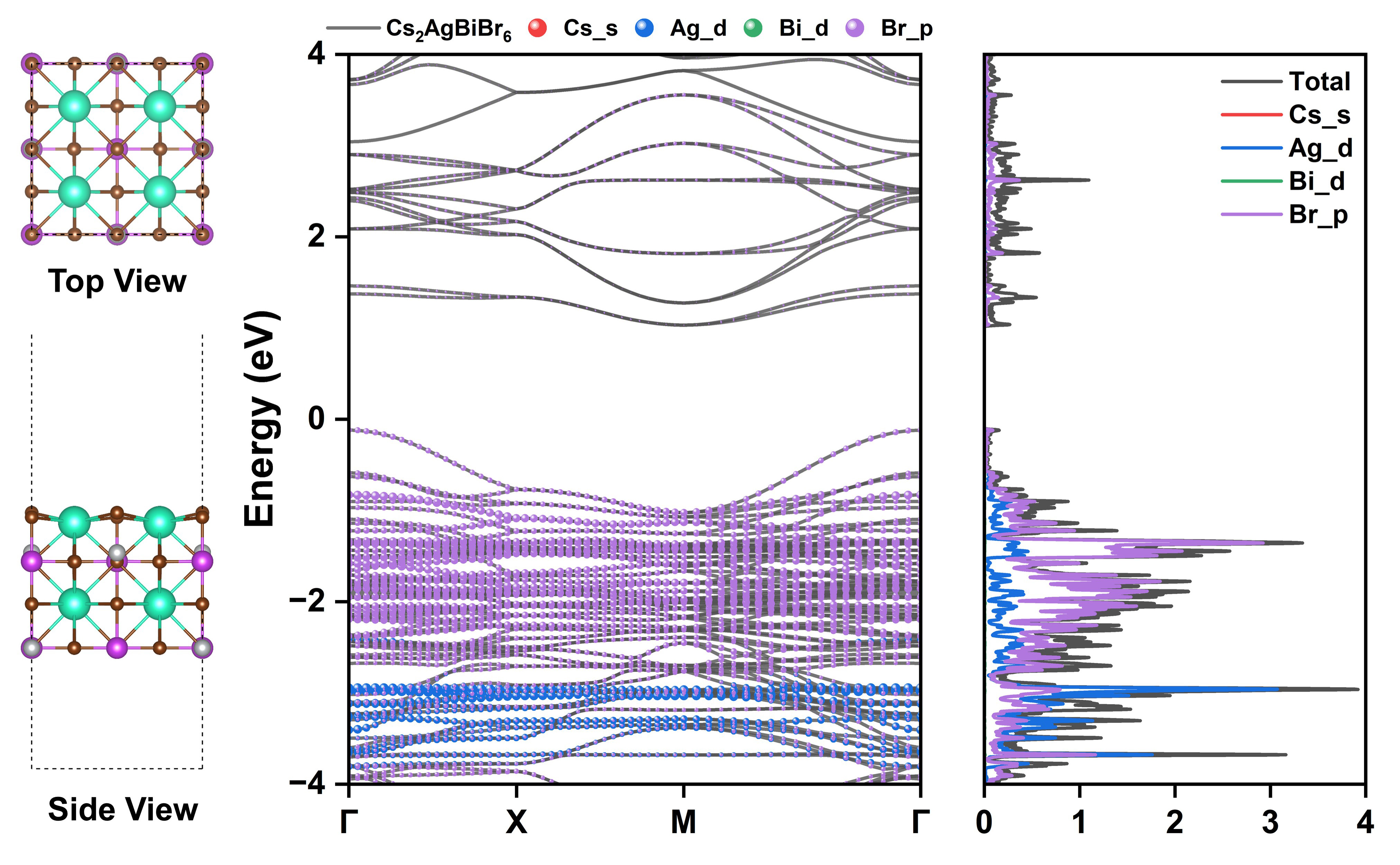


**Figure S1.** Intrinsic properties calculations of Cs_2_AgBiBr_6_.


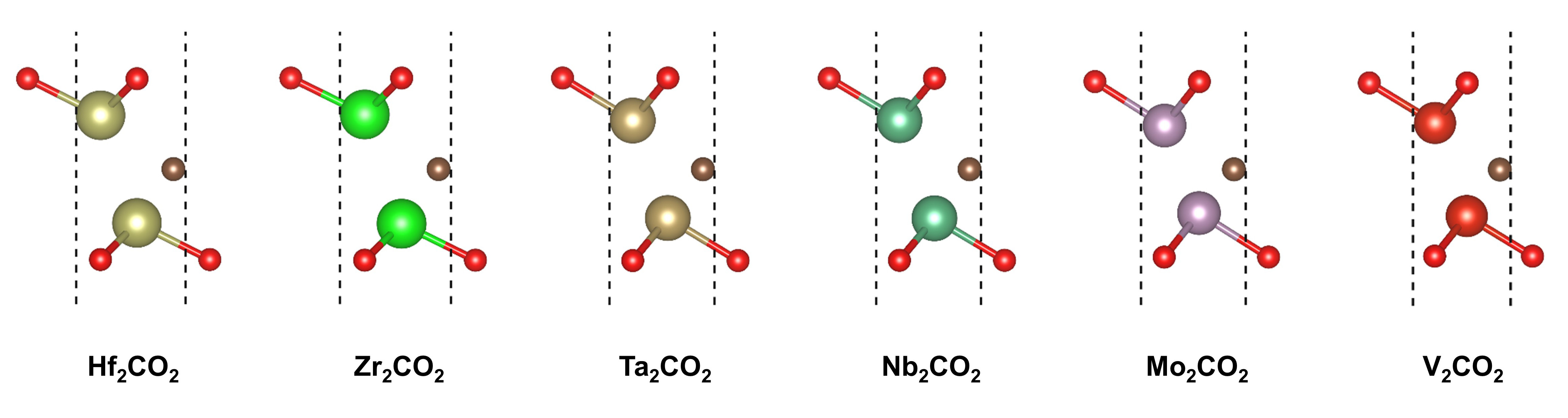


**Figure S2.** Crystal structures of Hf_2_CT_x_, Zr_2_CT_x_, Ta_2_CT_x_, Nb_2_CT_x_, Mo_2_CT_x_ and V_2_CT_x_.


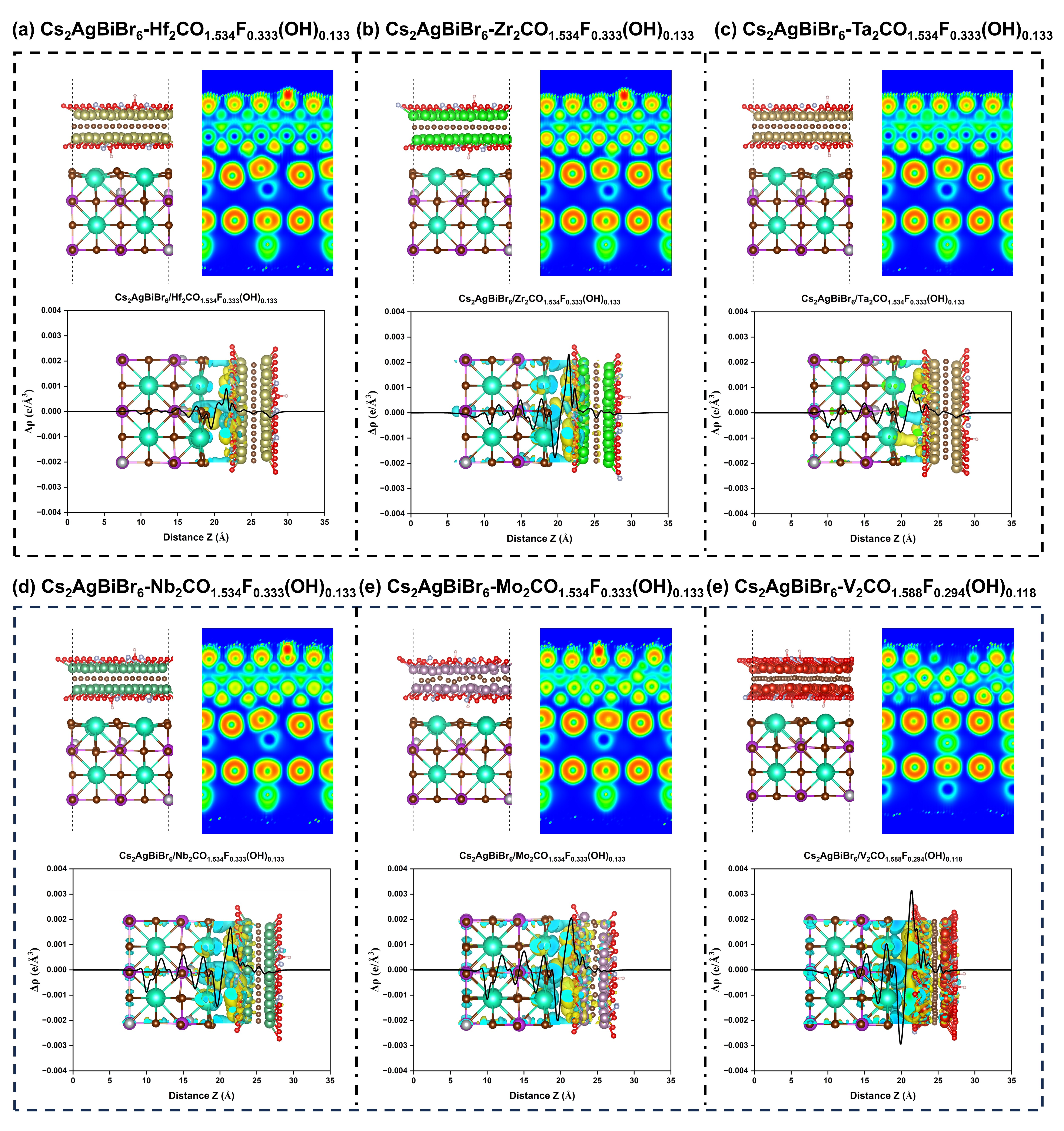


**Figure S3**. **Interfacial properties between Cs_2_AgBiBr_6_ and different M_2_X-type MXenes with mixed groups**. Crystal structure, electron localization function and difference charge density (Isosurface level is determined at the same level.) of (a) Cs_2_AgBiBr_6_/Hf_2_CO_1.534_F_0.333_(OH)_0.133_, (b) Cs_2_AgBiBr_6_/Zr_2_CO_1.534_F_0.333_(OH)_0.133_, (c) Cs_2_AgBiBr_6_/Ta_2_CO_1.534_F_0.333_(OH)_0.133_, (d) Cs_2_AgBiBr_6_/Nb_2_CO_1.534_F_0.333_(OH)_0.133_, (e) Cs_2_AgBiBr_6_/Mo_2_CO_1.534_F_0.333_(OH)_0.133_, (f) Cs_2_AgBiBr_6_/V_2_CO_1.588_F_0.294_(OH)_0.118_.


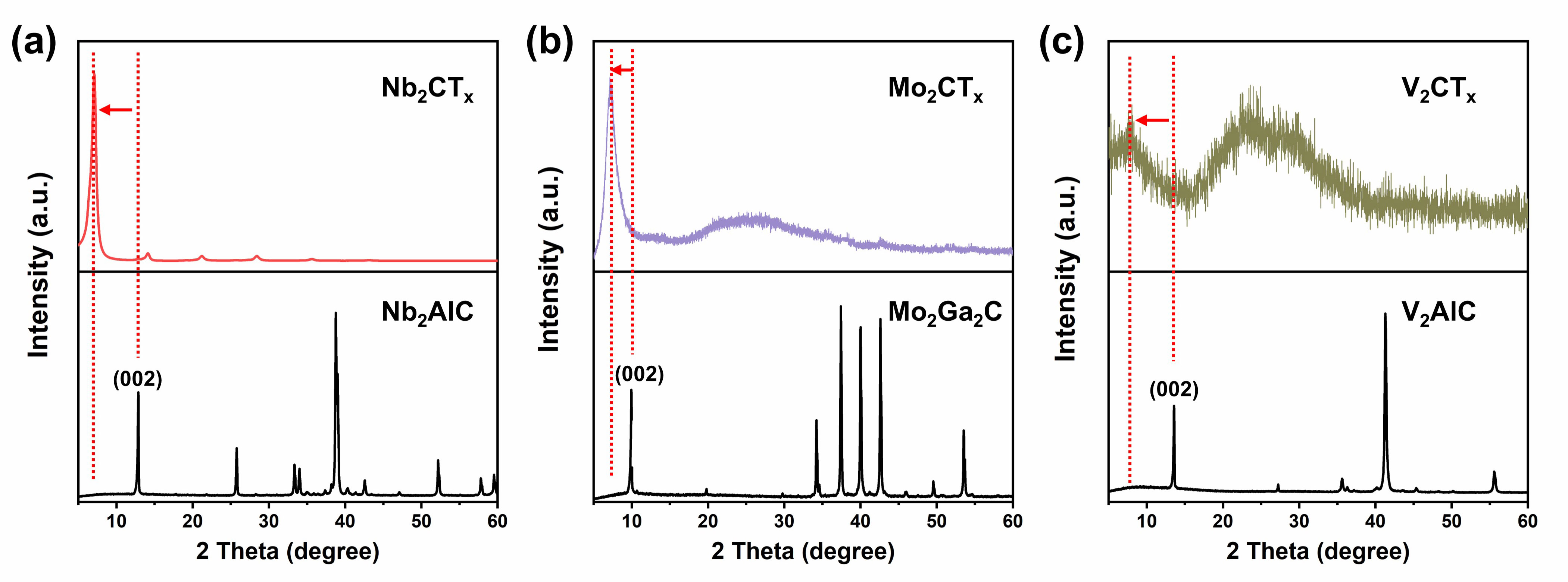


**Figure S4.** XRD patterns of (a) Nb_2_CT_x_, (b) Mo_2_CT_x_ and (c) V_2_CT_x_ three M_2_X-type MXenes before and after exfoliating.


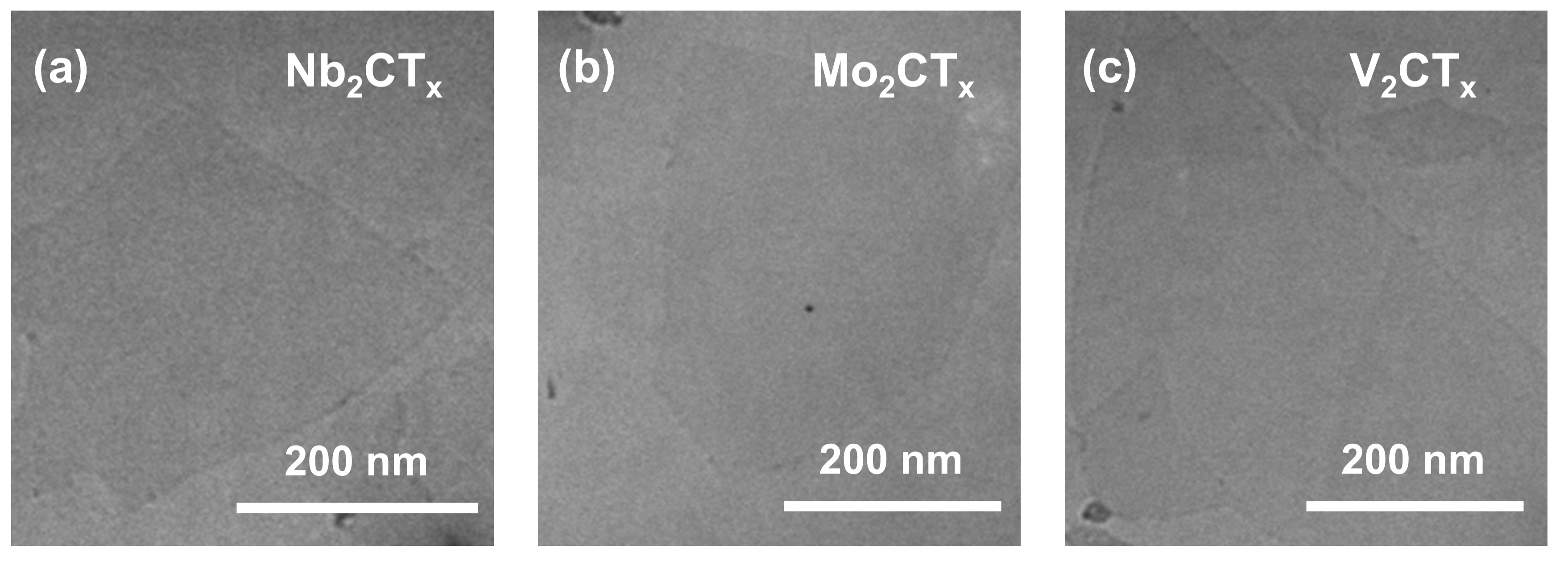


**Figure S5.** TEM images of (a) Nb_2_CT_x_, (b) Mo_2_CT_x_ and (c) V_2_CT_x_ nanosheets.


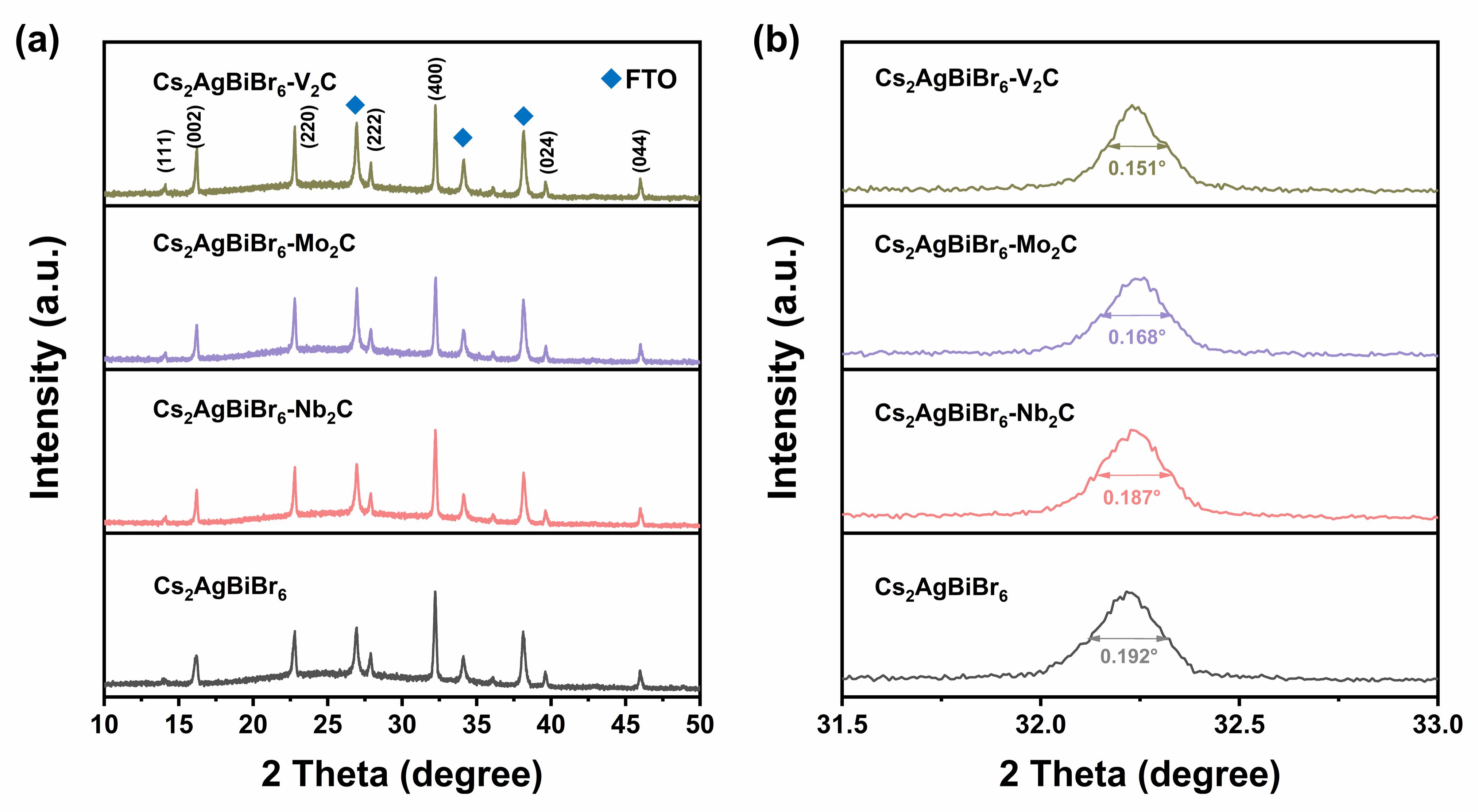


**Figure S6.** (a) XRD patterns of pristine Cs_2_AgBiBr_6_ and Cs_2_AgBiBr_6_ with different MXenes modification prepared by spin-coating on FTO substrates and (b) their FWHM of the (400) diffraction peaks at around 32.2°.


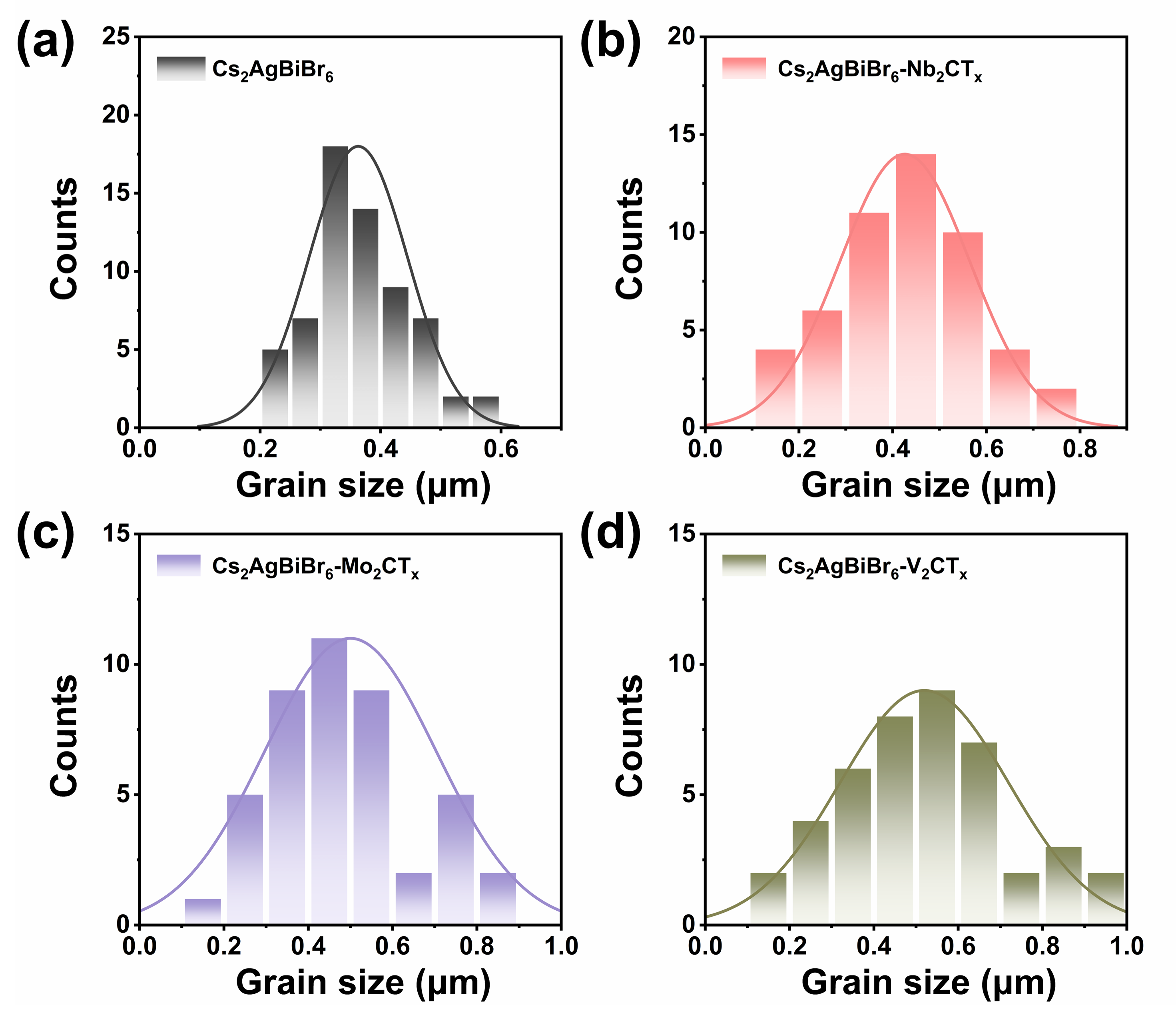


**Figure S7.** Grain size distributions of pristine Cs_2_AgBiBr_6_ film and those modified by (b) Nb_2_CT_x_, (c) Mo_2_CT_x_, and (d) V_2_CT_x_.


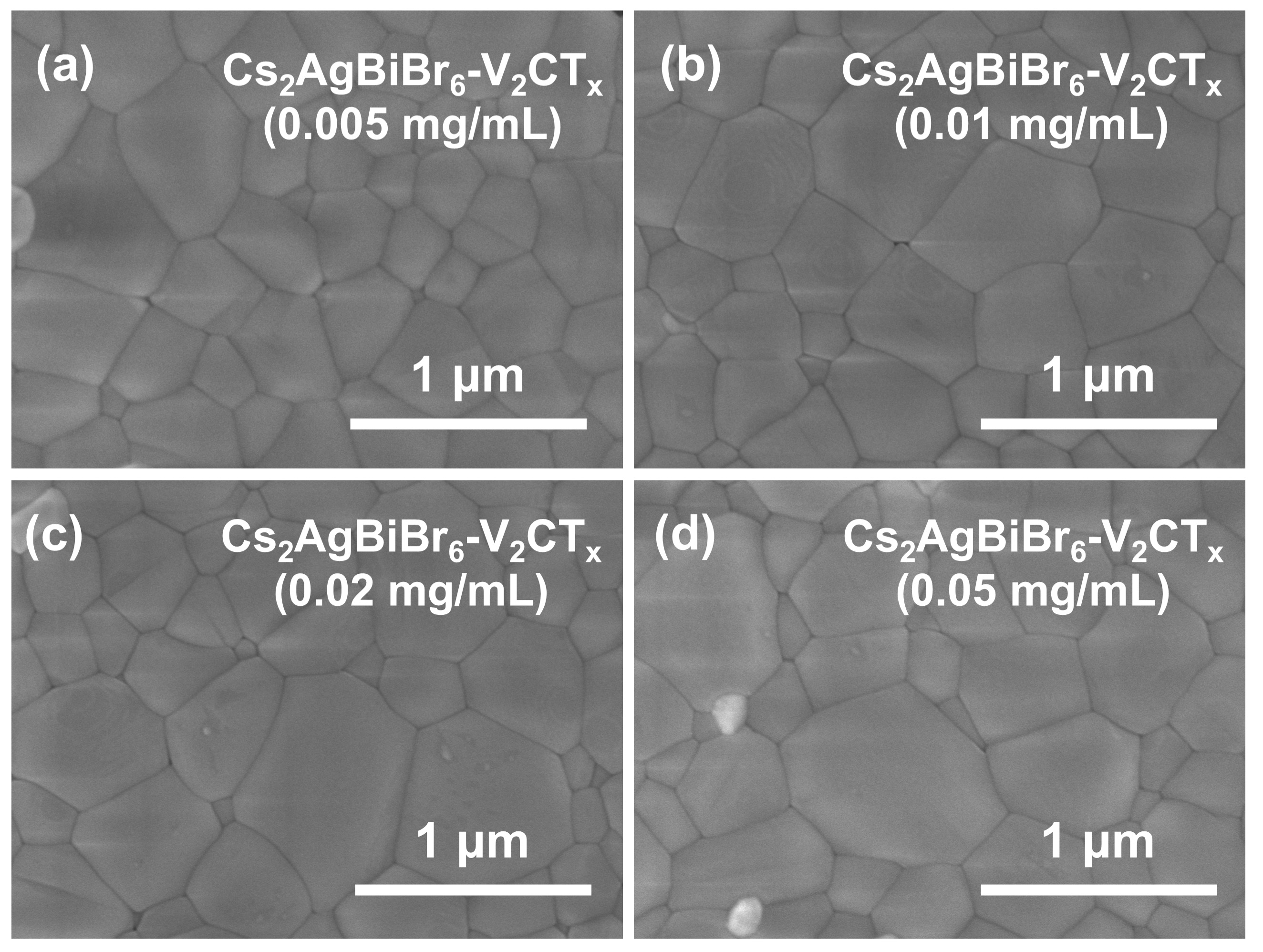


**Figure S8.** SEM images of Cs_2_AgBiBr_6_-V_2_CT_x_ films prepared by spin-coating with different concentrations.


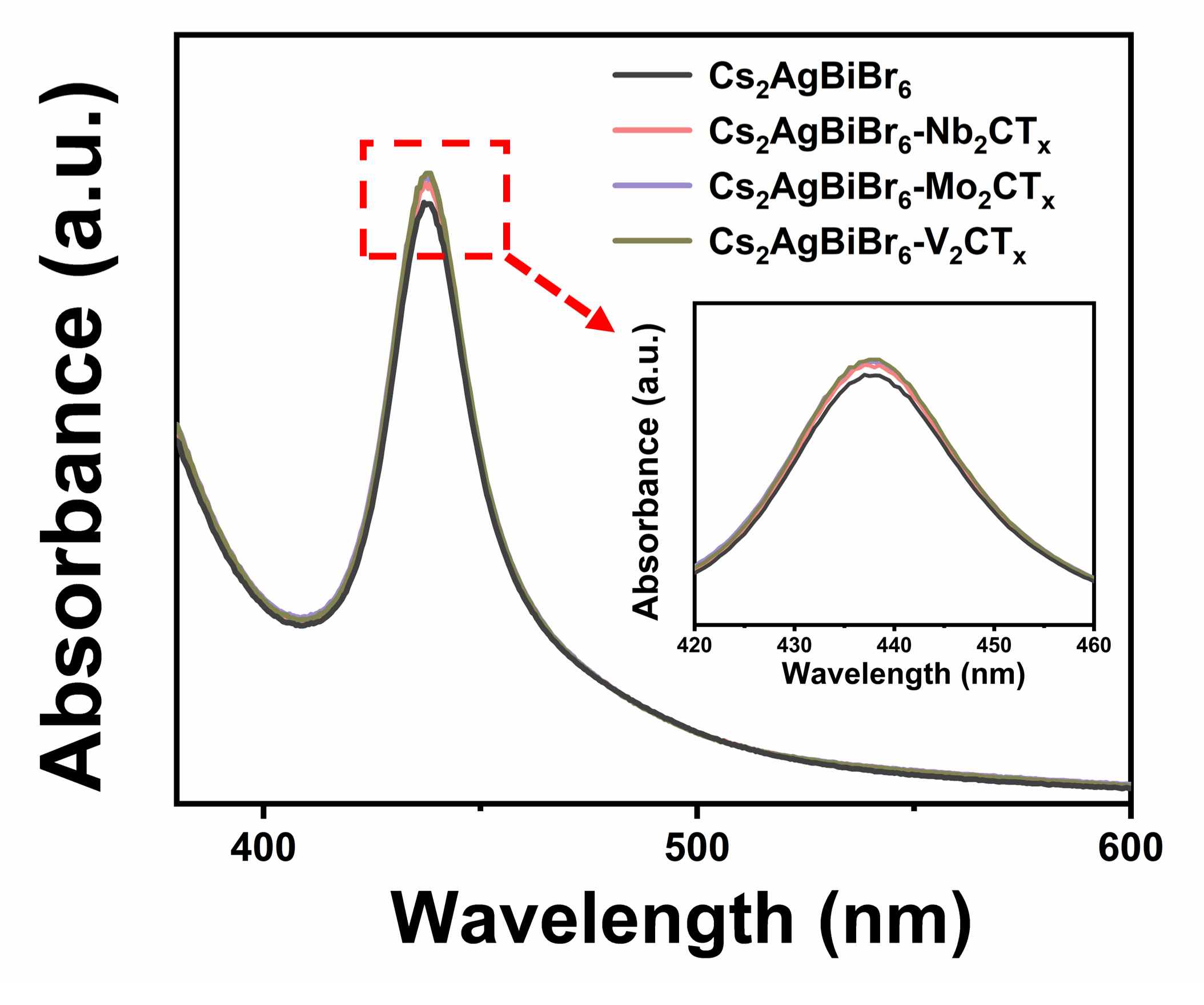


**Figure S9.** UV–vis absorption measurements of pristine Cs_2_AgBiBr_6_ and Cs_2_AgBiBr_6_ with different MXenes modification.


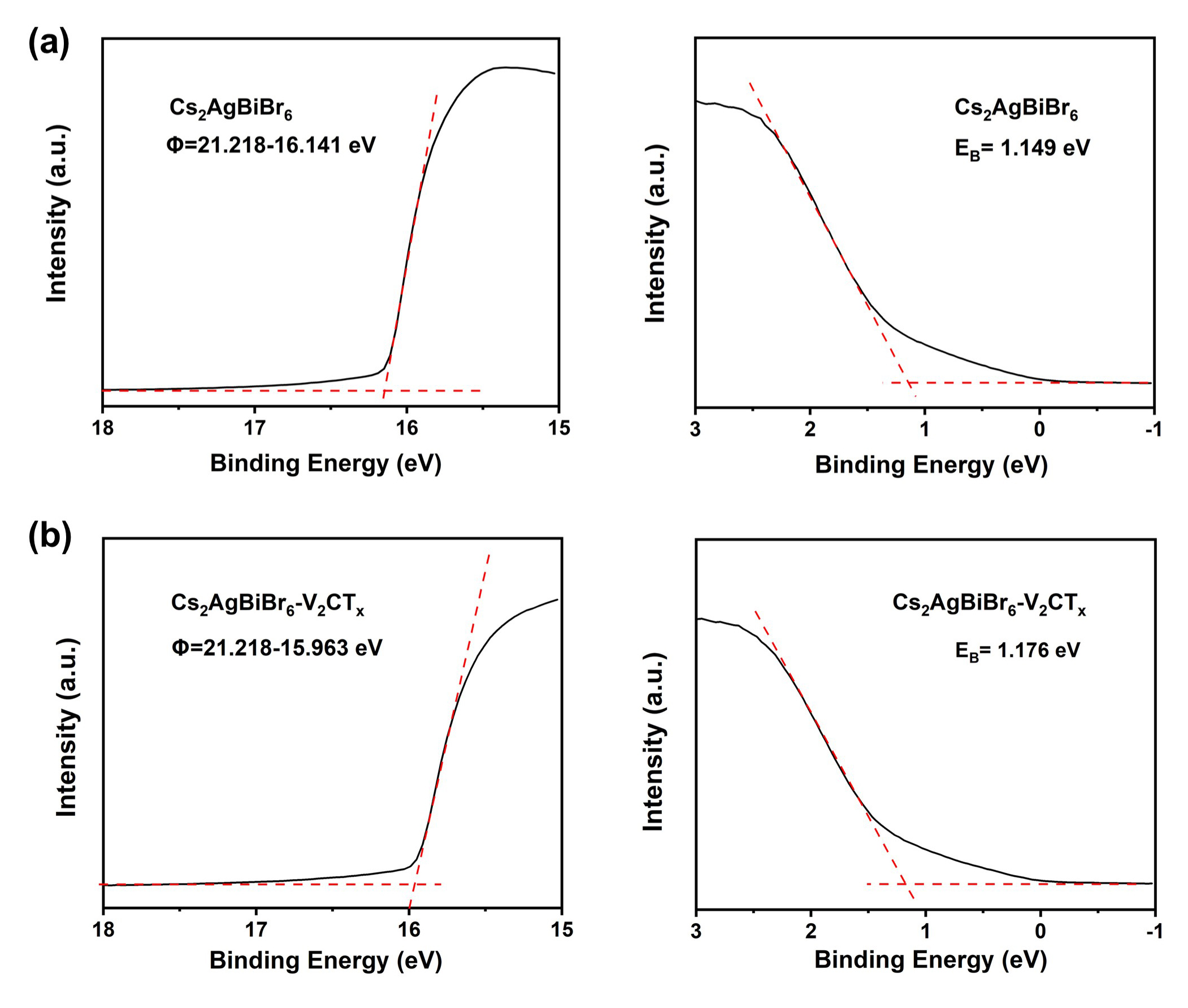


**Figure S10**. UPS spectra of (a) Cs_2_AgBiBr_6_ film and (b) Cs_2_AgBiBr_6_-V_2_CT_x_ film.


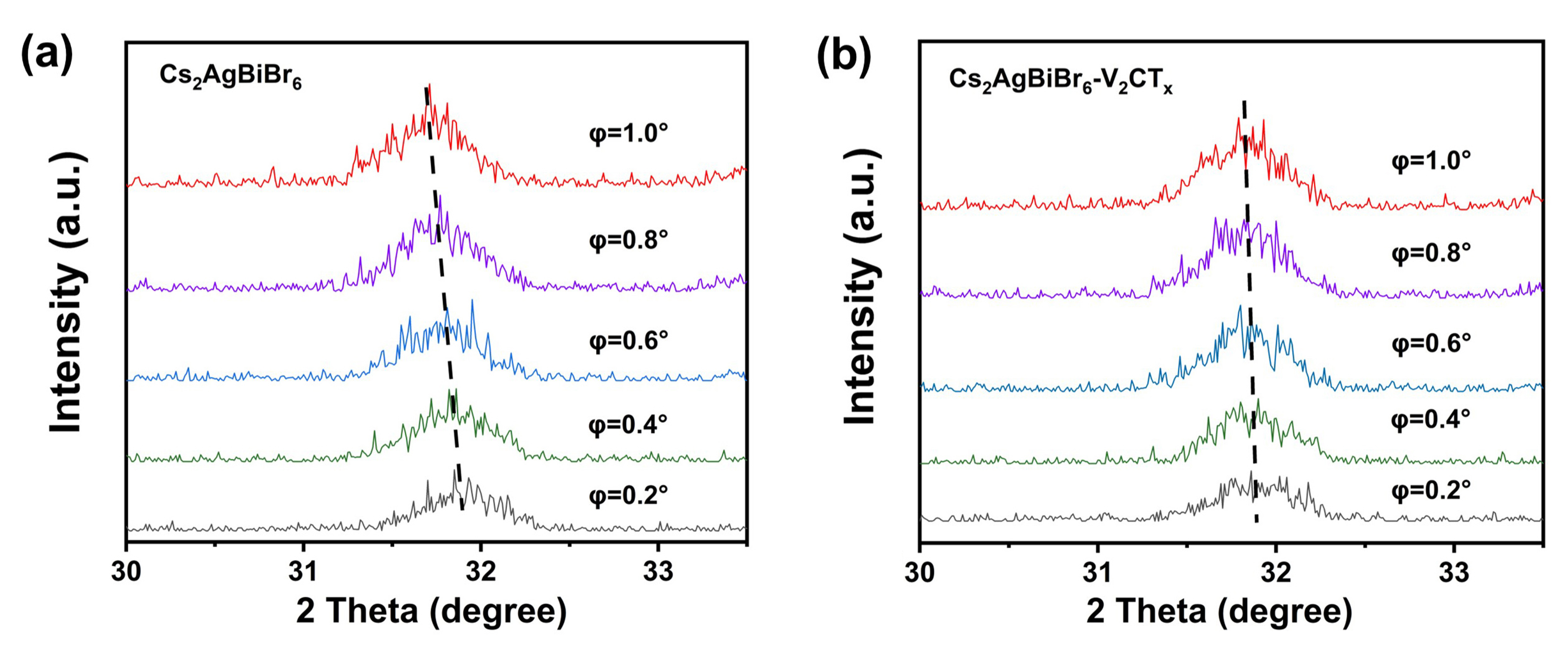


**Figure S11**. GIXRD patterns of (a) Cs_2_AgBiBr_6_ film and (b) Cs_2_AgBiBr_6_-V_2_CT_x_ film.
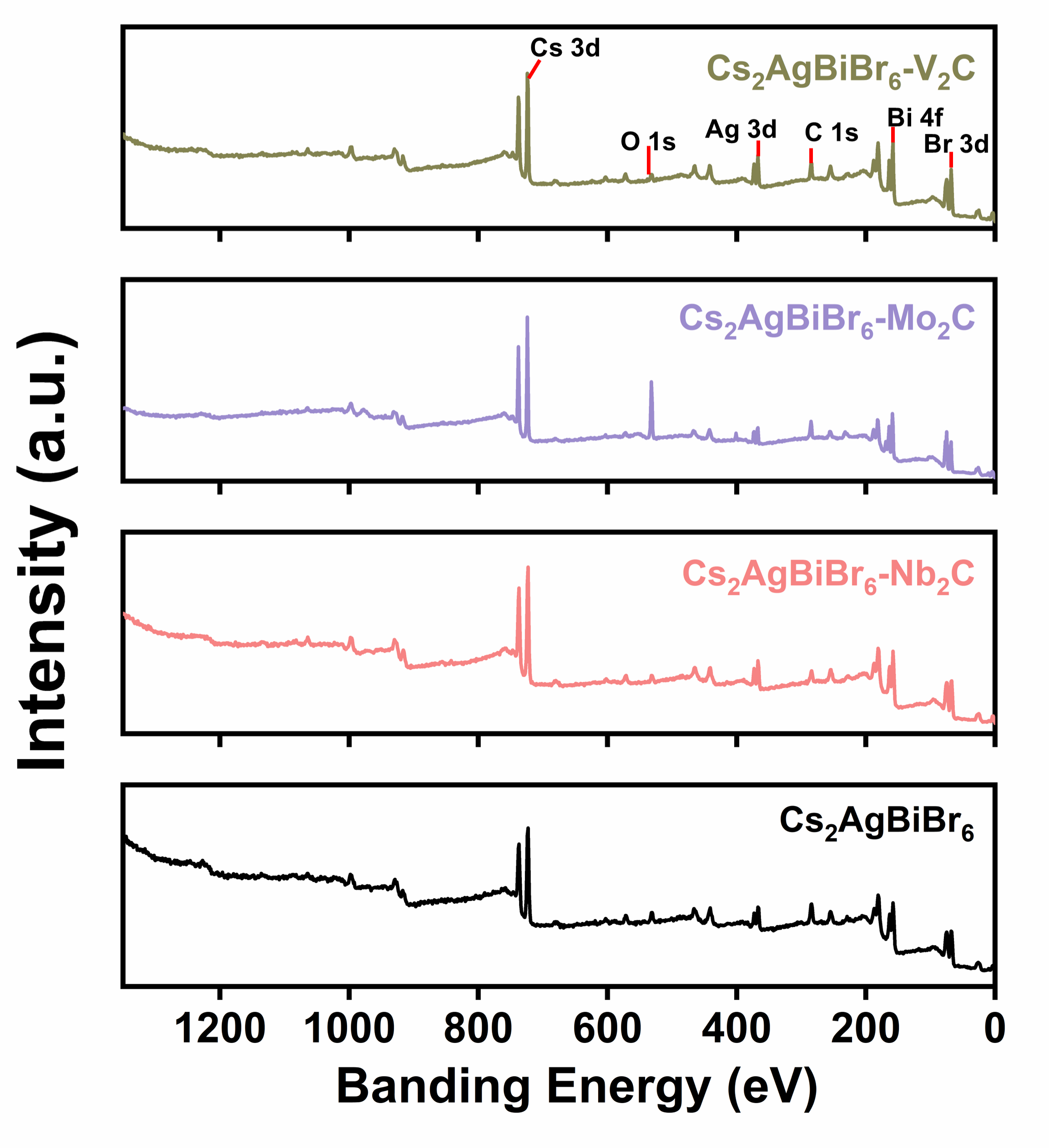


**Figure S12.** The full scan XPS spectra of pristine Cs_2_AgBiBr_6_ and Cs_2_AgBiBr_6_ with different MXenes modification.


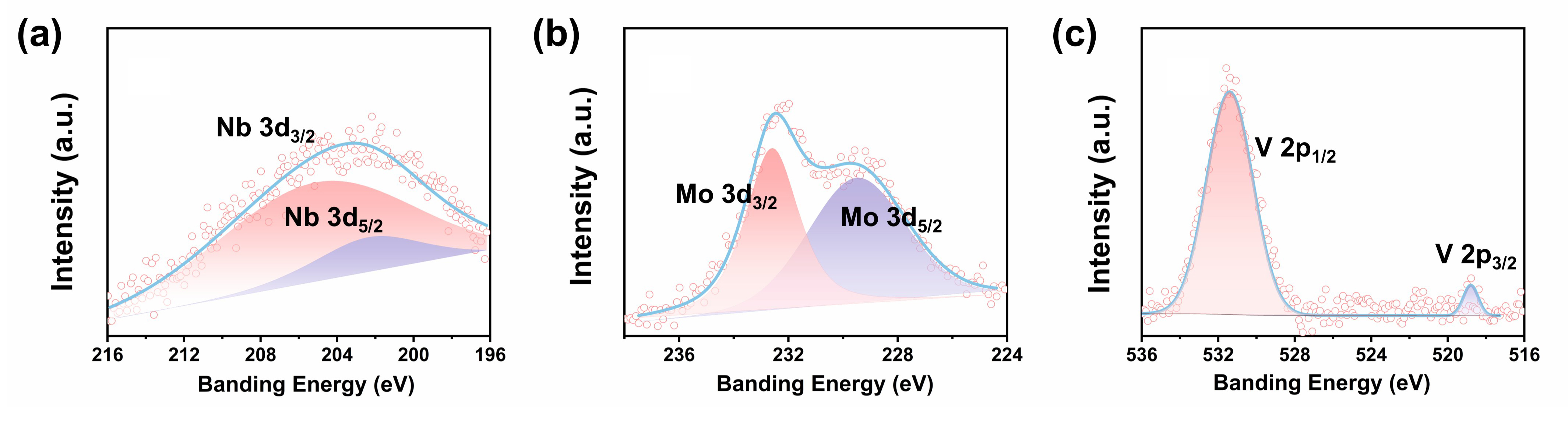


**Figure S13.** The fitted Nb 3d core-level spectra of Cs_2_AgBiBr_6_-Nb_2_CT_x_, fitted Mo 3d core-level spectra of Cs_2_AgBiBr_6_-Mo_2_CT_x_ and fitted V 2p core-level spectra of Cs_2_AgBiBr_6_-V_2_CT_x_.


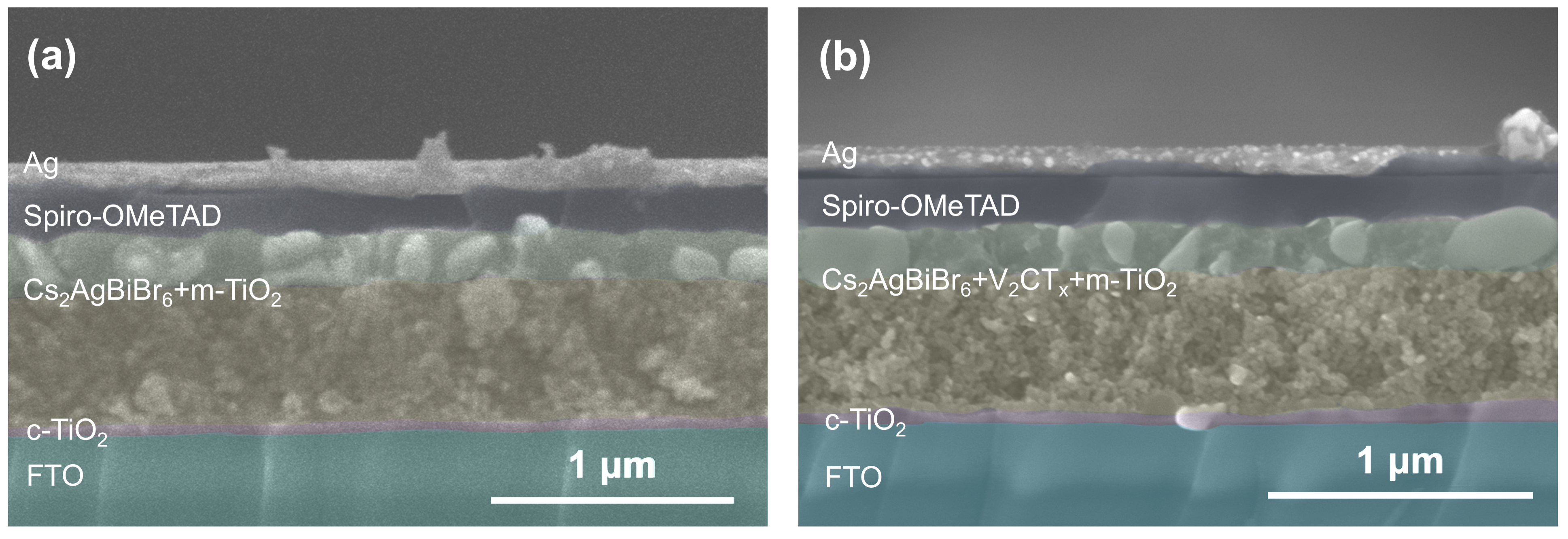


**Figure S14.** Cross-sectional SEM images of the devices based on pristine Cs_2_AgBiBr_6_ and Cs_2_AgBiBr_6_-V_2_CT_x_.


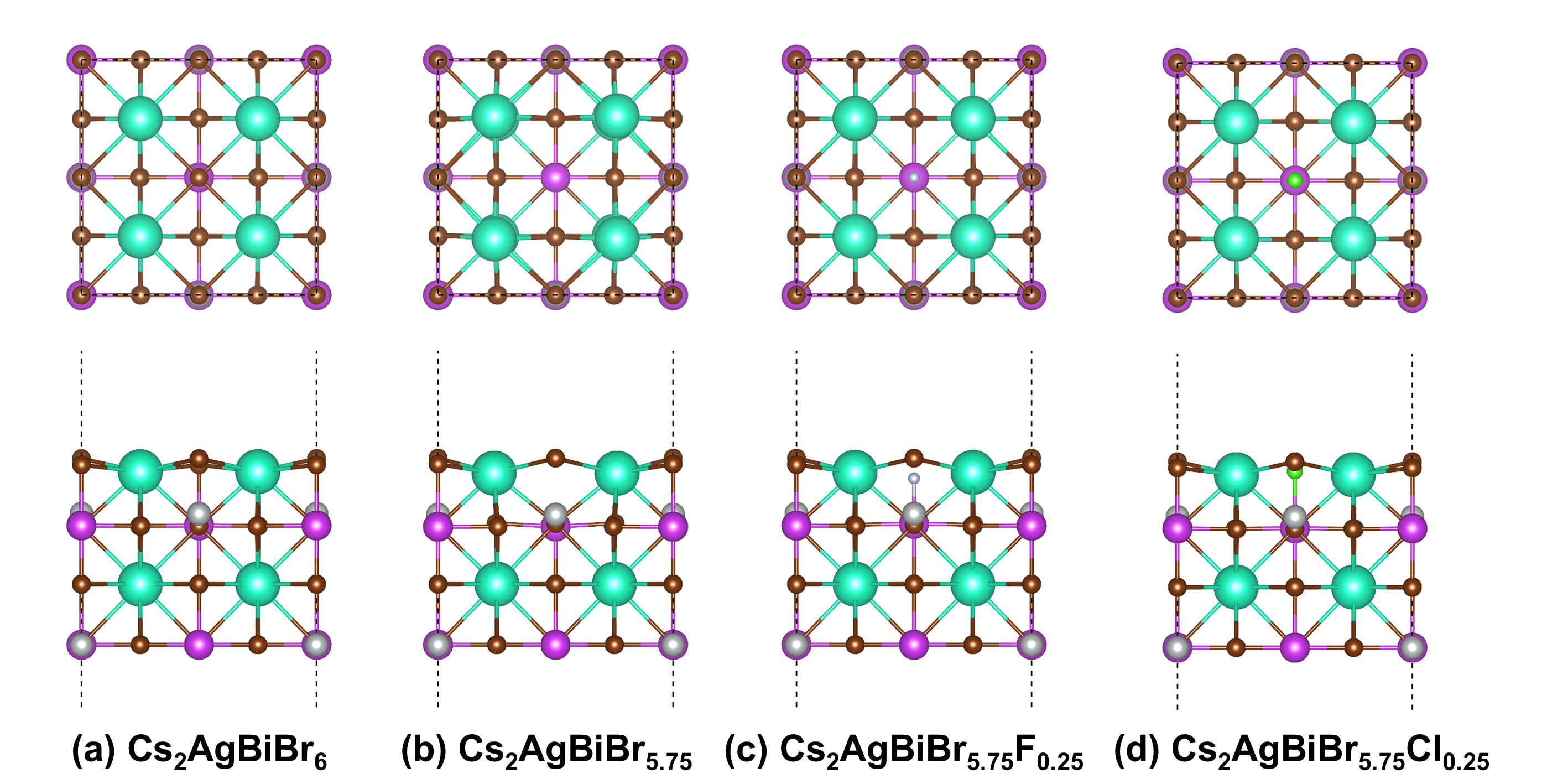


**Figure S15**. Top view and side view for (a) Cs_2_AgBiBr_6_, (b) Cs_2_AgBiBr_5.75_, (c) Cs_2_AgBiBr_5.75_F_0.25_, and (d) Cs_2_AgBiBr_5.75_Cl_0.25_.


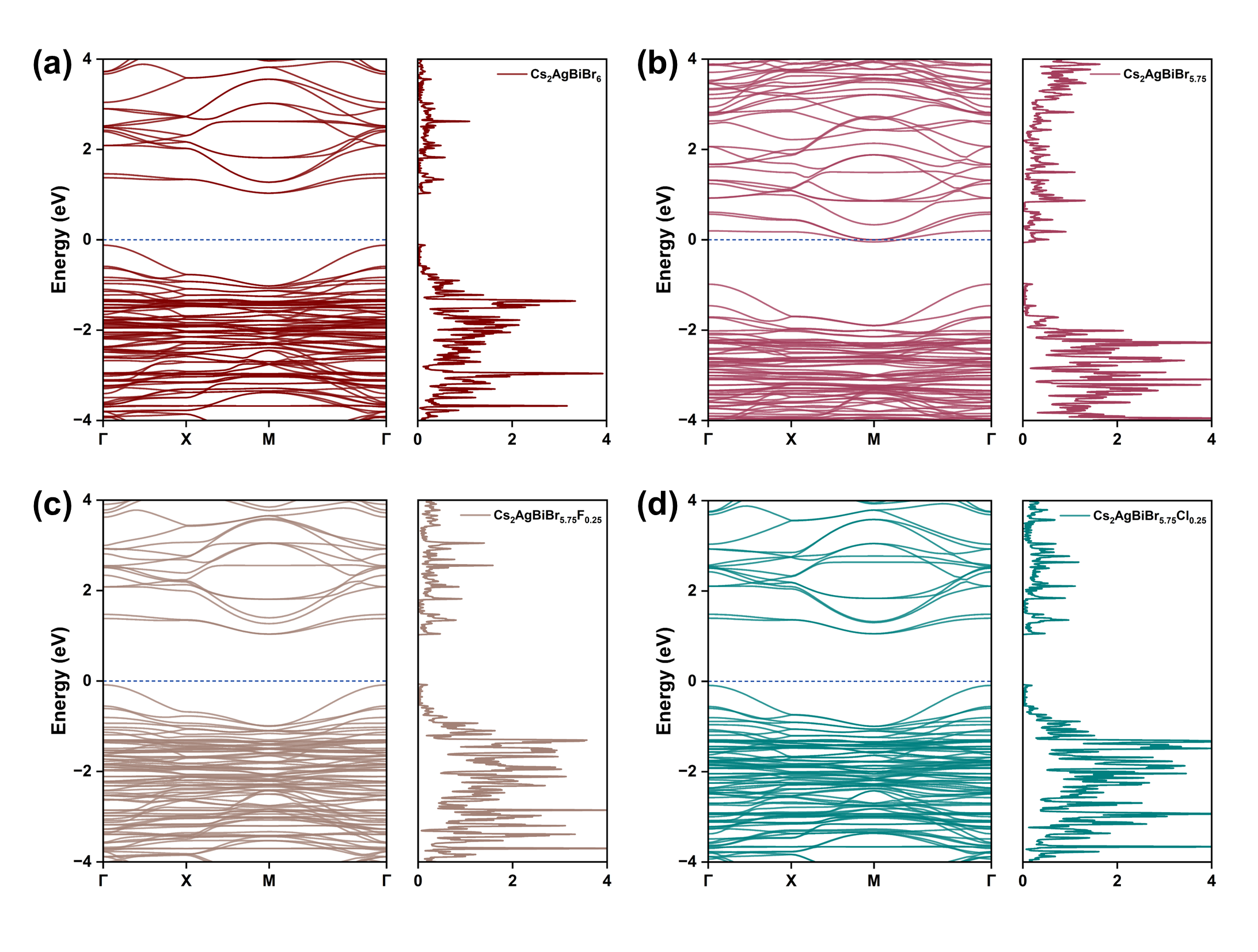


Figure S16. Band structures and density of states for (a) Cs_2_AgBiBr_6_, (b) Cs_2_AgBiBr_5.75_, (c) Cs_2_AgBiBr_5.75_F_0.25_, and (d) Cs_2_AgBiBr_5.75_Cl_0.25_.





**Figure S17.** *J-V* curves of devices based on Cs_2_AgBiBr_6_-Nb_2_CT_x_ with different concentrations.





**Figure S18.** J-V curves of devices based on Cs_2_AgBiBr_6_-Mo_2_CT_x_ with different concentrations.





**Figure S19.** Steady-state PL spectra of Cs_2_AgBiBr_6_-V_2_CT_x_ films with different concentrations.


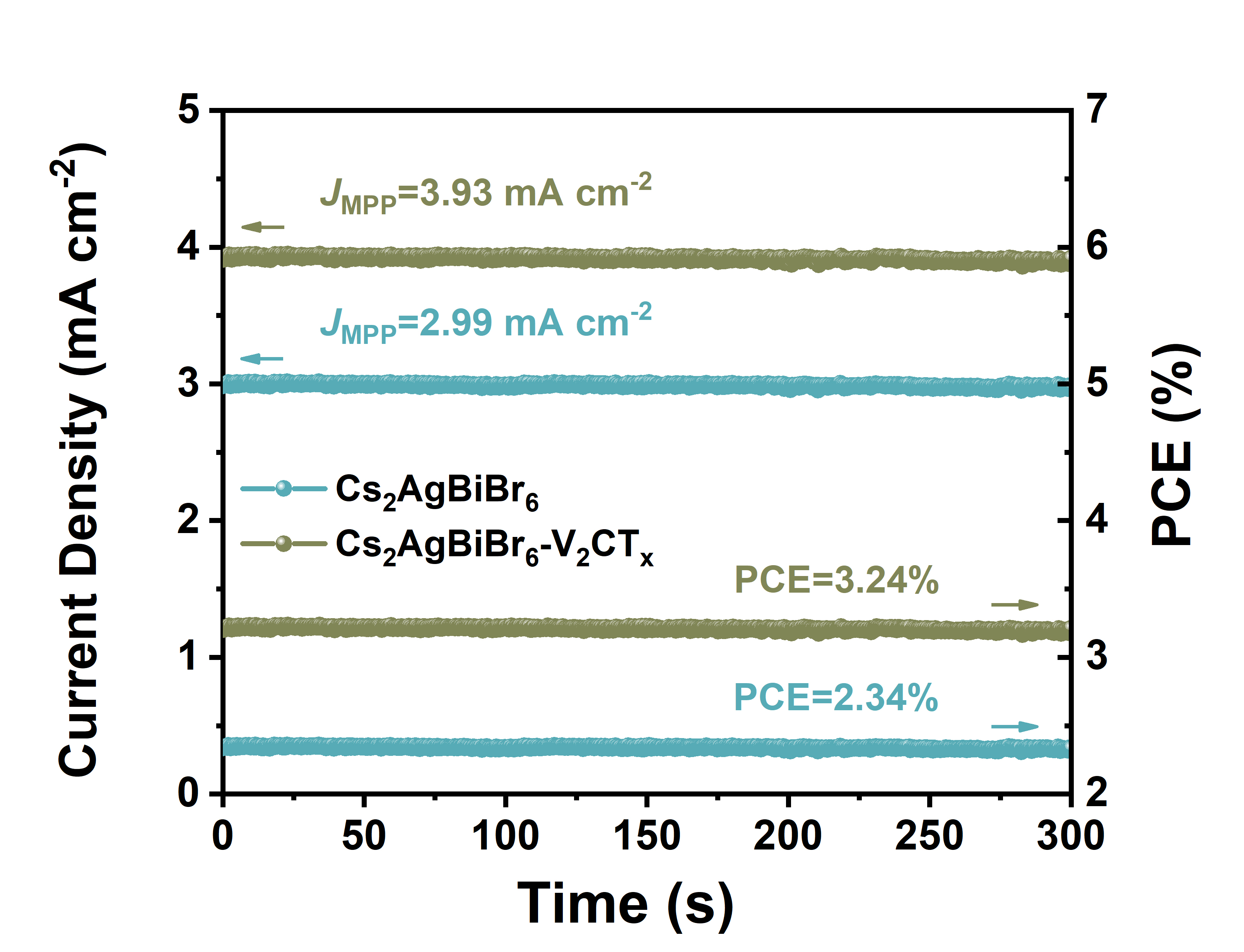


**Figure S20.** Steady-state power output of the devices based on pristine Cs_2_AgBiBr_6_ and Cs_2_AgBiBr_6_-V_2_CT_x_.


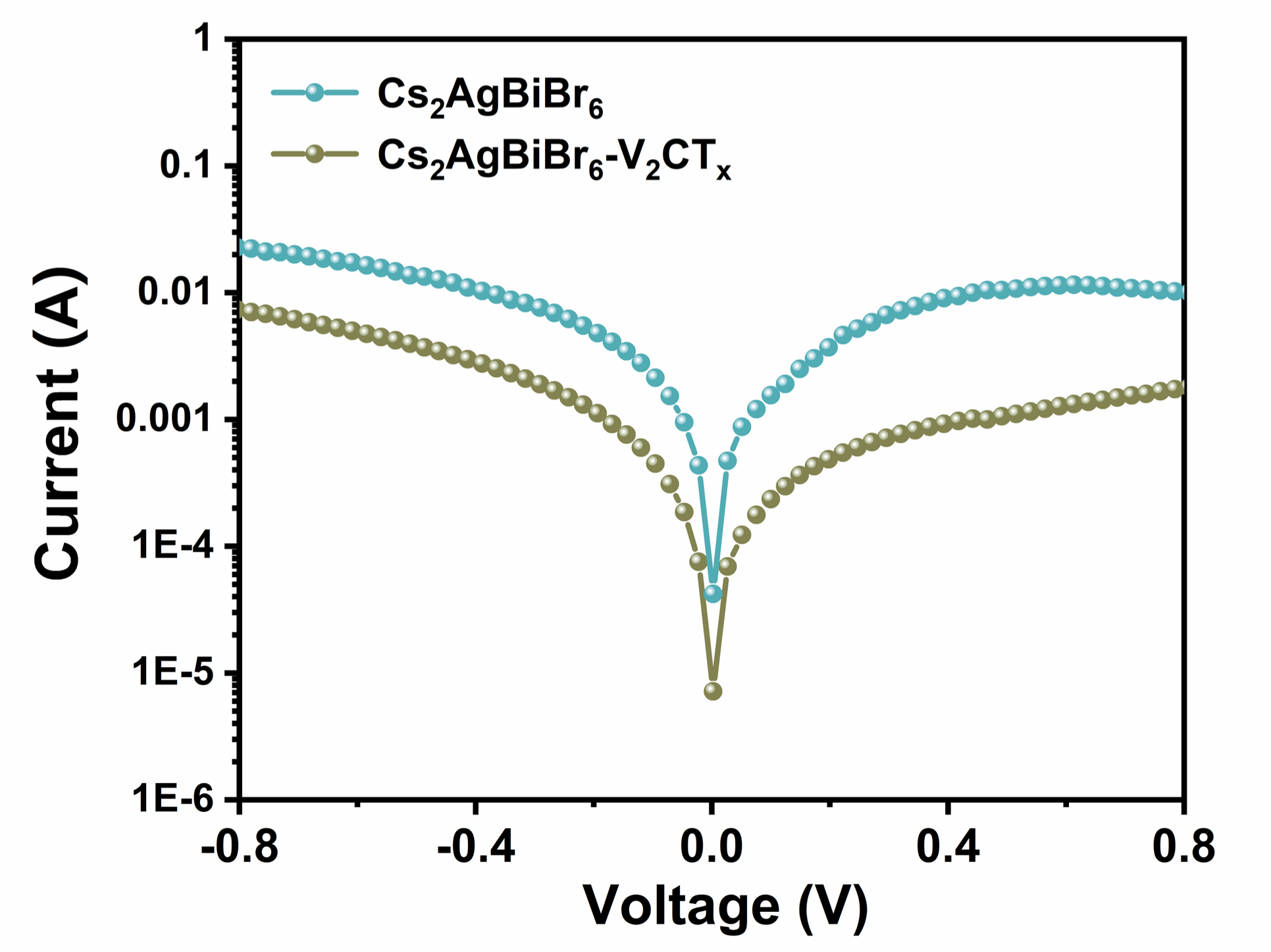


**Figure S21.** Dark *J−V* measurements of PSCs based on pristine Cs_2_AgBiBr_6_ and Cs_2_AgBiBr_6_-V_2_CT_x_.





**Figure S22.** *J_sc_* changes of the devices based on pristine Cs_2_AgBiBr_6_ and Cs_2_AgBiBr_6_-V_2_CT_x_ with different incident light intensity.





**Figure S23.** *V_oc_* changes of the devices based on pristine Cs_2_AgBiBr_6_ and Cs_2_AgBiBr_6_-V_2_CT_x_ with different incident light intensity.


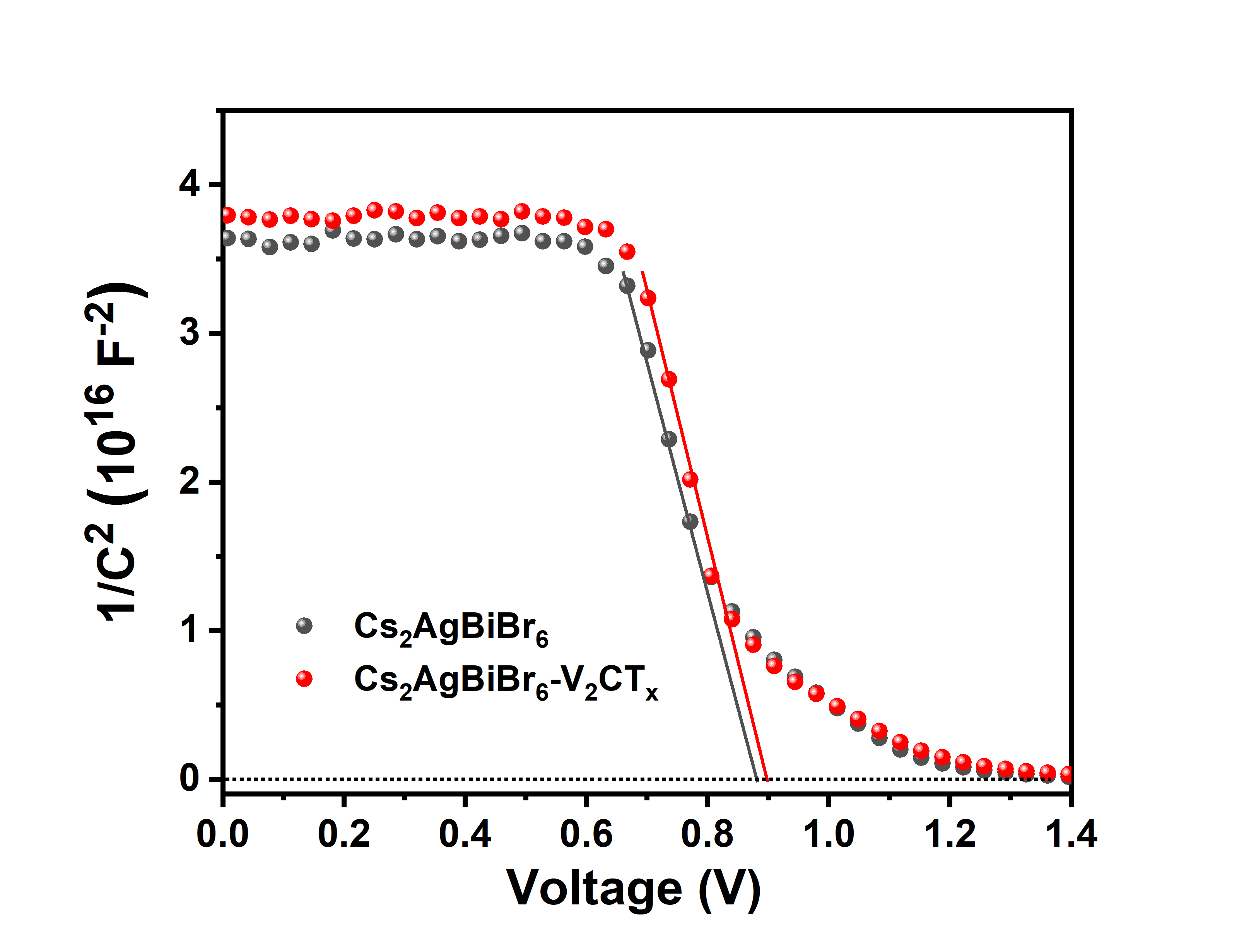


Figure S24. Mott−Schottky plots of the devices based on pristine Cs_2_AgBiBr_6_ and Cs_2_AgBiBr_6_-V_2_CT_x_.


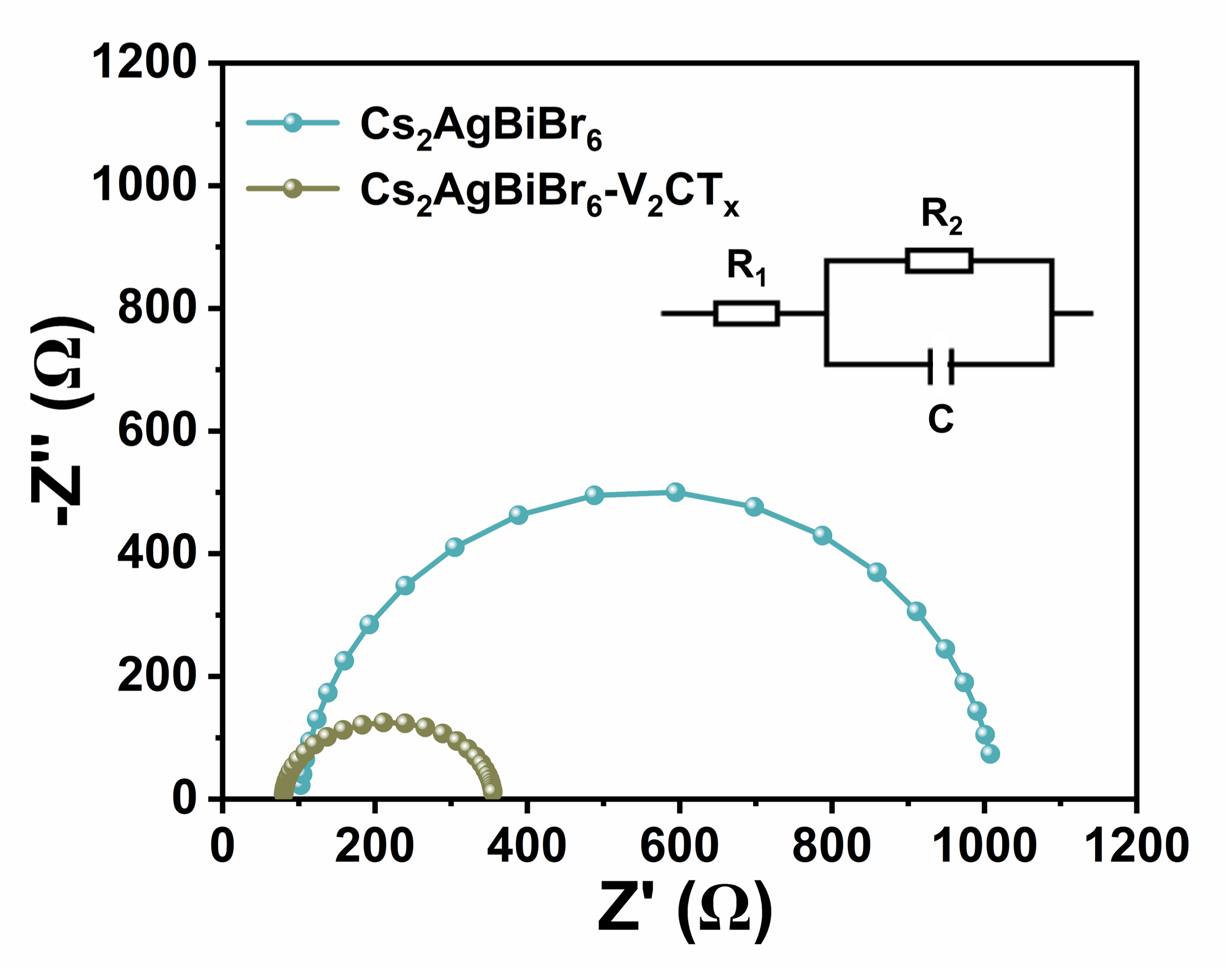


**Figure S25.** Nyquist plots of the devices based on Cs_2_AgBiBr_6_ and Cs_2_AgBiBr_6_-V_2_CT_x_, where the spectra inset refers to the corresponding equivalent circuit.


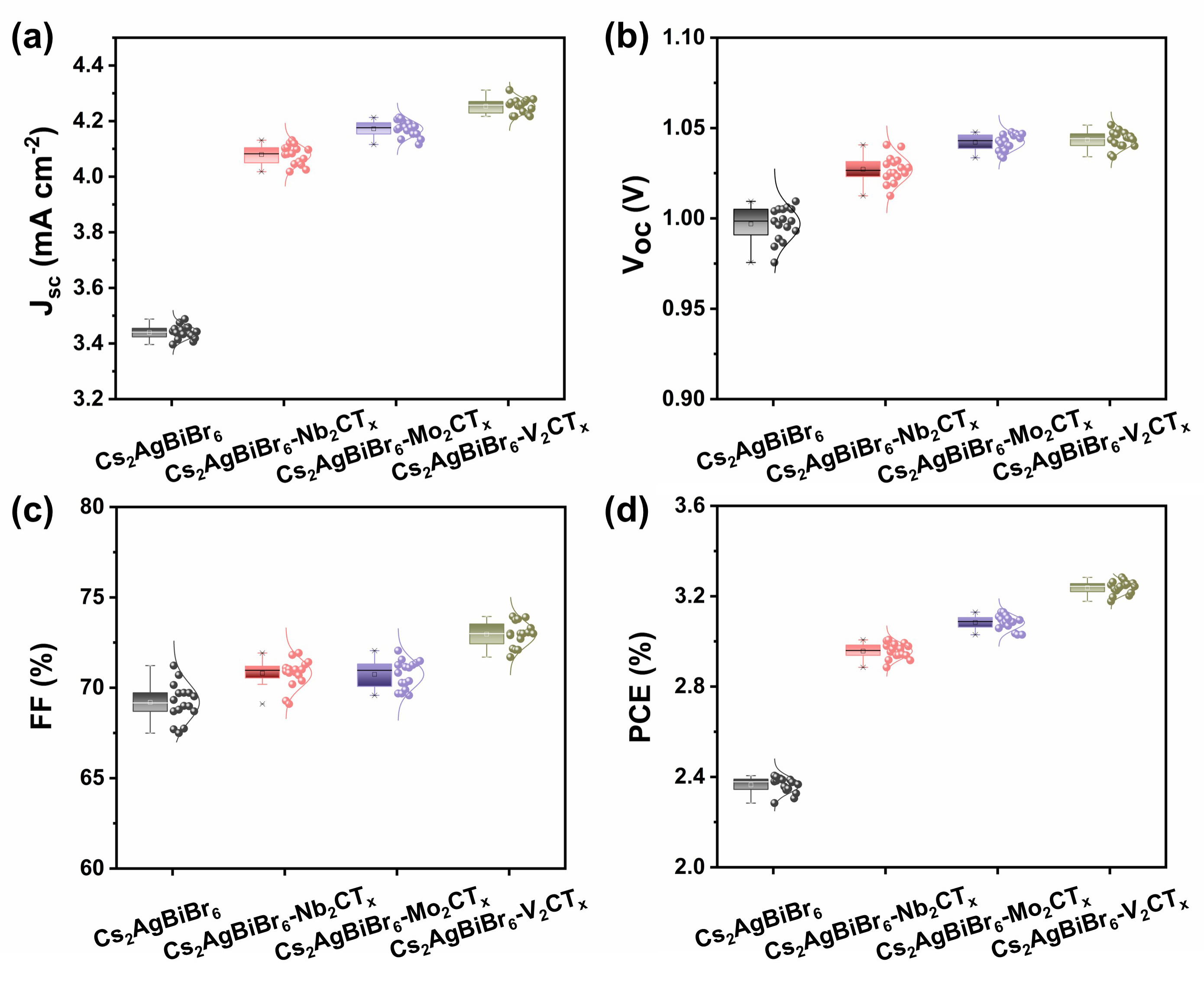


**Figure S26.** Box diagrams of the photovoltaic performance parameters of the devices based on pristine Cs_2_AgBiBr_6_ and Cs_2_AgBiBr_6_ with different MXenes modification.

**Table S1.** Intrinsic properties predictions of Cs_2_AgBiBr_6_

|  | **Vacuum level (eV)** | **Fermi level (eV)** | **VBM**  **(eV)** | |  |
| --- | --- | --- | --- | --- | --- |
| Cs_2_AgBiBr_6_ | 2.337 | -3.180 | -3.299 | |  |
|  | **CBM (eV)** | **EA (eV)** | **IP (eV)** | **W (eV)** | |
|  | -2.148 | 4.485 | 5.636 | 5.517 | |

Table S2. The layer spacing of the heterojunction models before and after geometric optimization.

| Species | Initial layer spacing (Å) | Final layer spacing (Å) |
| --- | --- | --- |
| Cs_2_AgBiBr_6_-Hf_2_CO_2_ | 3.000 | 3.234 |
| Cs_2_AgBiBr_6_-Zr_2_CO_2_ | 3.000 | 3.160 |
| Cs_2_AgBiBr_6_-Ta_2_CO_2_ | 3.000 | 3.288 |
| Cs_2_AgBiBr_6_-Nb_2_CO_2_ | 3.000 | 3.227 |
| Cs_2_AgBiBr_6_-Mo_2_CO_2_ | 3.000 | 2.998 |
| Cs_2_AgBiBr_6_-V_2_CO_2_ | 3.000 | 2.911 |

**Table S3**. Electron, hole Schottky barriers predicted by the Schottky-Mott model.

($\boldsymbol{\phi}^{\boldsymbol{ⅇ}}\boldsymbol{=W-}\boldsymbol{E}_{\boldsymbol{ea}}$**，**$\boldsymbol{\phi}^{\boldsymbol{h}}\boldsymbol{=}\boldsymbol{E}_{\boldsymbol{ip}}\boldsymbol{-W}$)

| MXenes | **W (eV)** | $\boldsymbol{\phi}^{\boldsymbol{ⅇ}}$ **(eV)** | $\boldsymbol{\phi}^{\boldsymbol{h}}$ **(eV)** |
| --- | --- | --- | --- |
| Hf_2_CO_2_ | 5.303 | 0.818 | 0.333 |
| Zr_2_CO_2_ | 5.327 | 0.842 | 0.309 |
| Ta_2_CO_2_ | 5.435 | 0.950 | 0.201 |
| Nb_2_CO_2_ | 5.703 | 1.218 | -0.067 |
| Mo_2_CO_2_ | 6.348 | 1.863 | -0.712 |
| V_2_CO_2_ | 6.626 | 2.141 | -0.990 |

**Table S4.** Calculation data of heterojunctions predicted by the Schottky-Mott model.

| MXenes | **Mismatch degree (%)** | **Electron Transfer (e/Å^2^)** | **P-type barrier (eV)** |
| --- | --- | --- | --- |
| Cs_2_AgBiBr_6_-Hf_2_CT_x_ | 2.806 | 0.00071 | -0.005 |
| Cs_2_AgBiBr_6_-Zr_2_CT_x_ | 4.016 | 0.00129 | -0.101 |
| Cs_2_AgBiBr_6_-Ta_2_CT_x_ | 1.130 | 0.00167 | -0.259 |
| Cs_2_AgBiBr_6_-Nb_2_CT_x_ | 1.242 | 0.00231 | -0.262 |
| Cs_2_AgBiBr_6_-Mo_2_CT_x_ | 2.866 | 0.00342 | -0.263 |
| Cs_2_AgBiBr_6_-V_2_CT_x_ | 4.105 | 0.00468 | -0.375 |

**Table S5**. The layer spacing of the heterojunction models with mixed functional groups before and after geometric optimization.

| Species | | Initial layer spacing (Å) | | Final layer spacing (Å) | |
| --- | --- | --- | --- | --- | --- |
| Cs_2_AgBiBr_6_-Hf_2_CO_1.534_F_0.333_(OH)_0.133_ | 3.000 | | 3.140 | |  |
| Cs_2_AgBiBr_6_-Zr_2_CO_1.534_F_0.333_(OH)_0.133_ | 3.000 | | 3.058 | |  |
| Cs_2_AgBiBr_6_-Ta_2_CO_1.534_F_0.333_(OH)_0.133_ | 3.000 | | 3.220 | |  |
| Cs_2_AgBiBr_6_-Nb_2_CO_1.534_F_0.333_(OH)_0.133_ | 3.000 | | 2.995 | |  |
| Cs_2_AgBiBr_6_-Mo_2_CO_1.534_F_0.333_(OH)_0.133_ | 3.000 | | 2.909 | |  |
| Cs_2_AgBiBr_6_-V_2_CO_1.588_F_0.294_(OH)_0.118_ | 3.000 | | 2.854 | |  |

**Table S6.** The photovoltaic performance parameters of the devices based on pristine Cs_2_AgBiBr_6_ and those with different MXenes modification.

|  | **Scan direction** | ***V_oc_* (V)** | ***J_sc_* (mA cm^-2^)** | **FF** | **PCE (%)** |
| --- | --- | --- | --- | --- | --- |
| Cs_2_AgBiBr_6_ | Reverse | 1.004 | 3.45 | 0.691 | 2.38 |
|  | Forward | 0.966 | 3.36 | 0.662 | 2.15 |
| Cs_2_AgBiBr_6_-Nb_2_CT_x_ | Reverse | 1.040 | 4.07 | 0.708 | 2.99 |
|  | Forward | 1.027 | 4.03 | 0.680 | 2.81 |
| Cs_2_AgBiBr_6_-Mo_2_CT_x_ | Reverse | 1.043 | 4.16 | 0.717 | 3.12 |
|  | Forward | 1.026 | 4.09 | 0.695 | 2.92 |
| Cs_2_AgBiBr_6_-V_2_CT_x_ | Reverse | 1.041 | 4.26 | 0.733 | 3.25 |
|  | Forward | 1.024 | 4.24 | 0.713 | 3.09 |

**Table S7.** The photovoltaic performance parameters of the devices based on Cs_2_AgBiBr_6_-V_2_CT_x_ with different concentrations.

|  | ***V_oc_* (V)** | ***J*_sc_(mA cm^-2^)** | **FF** | **PCE (%)** |
| --- | --- | --- | --- | --- |
| Cs_2_AgBiBr_6_ | 1.004 | 3.45 | 0.691 | 2.38 |
| Cs_2_AgBiBr_6_-V_2_CT_x_ (0.005 mg/mL) | 1.026 | 3.63 | 0.701 | 2.61 |
| Cs_2_AgBiBr_6_-V_2_CT_x_ (0.01 mg/mL) | 1.040 | 3.88 | 0.697 | 2.81 |
| Cs_2_AgBiBr_6_-V_2_CT_x_ (0.02 mg/mL) | 1.041 | 4.26 | 0.733 | 3.25 |
| Cs_2_AgBiBr_6_-V_2_CT_x_ (0.05 mg/mL) | 1.043 | 4.18 | 0.701 | 3.05 |

**Table S8.** The photovoltaic performance parameters of the devices based on Cs_2_AgBiBr_6_-Nb_2_CT_x_ with different concentrations.

|  | ***V_oc_* (V)** | ***J*_sc_(mA cm^-2^)** | **FF** | **PCE (%)** |
| --- | --- | --- | --- | --- |
| Cs_2_AgBiBr_6_ | 1.004 | 3.45 | 0.691 | 2.38 |
| Cs_2_AgBiBr_6_-Nb_2_CT_x_ (0.005 mg/mL) | 1.014 | 3.59 | 0.690 | 2.51 |
| Cs_2_AgBiBr_6_-Nb_2_CT_x_ (0.01 mg/mL) | 1.026 | 3.79 | 0.701 | 2.73 |
| Cs_2_AgBiBr_6_-Nb_2_CT_x_ (0.02 mg/mL) | 1.040 | 4.07 | 0.708 | 2.99 |
| Cs_2_AgBiBr_6_-Nb_2_CT_x_ (0.05 mg/mL) | 1.030 | 4.08 | 0.697 | 2.93 |

**Table S9.** The photovoltaic performance parameters of the devices based on Cs_2_AgBiBr_6_-Mo_2_CT_x_ with different concentrations.

|  | ***V_oc_* (V)** | ***J*_sc_(mA cm^-2^)** | **FF** | **PCE (%)** |
| --- | --- | --- | --- | --- |
| Cs_2_AgBiBr_6_ | 1.004 | 3.45 | 0.691 | 2.38 |
| Cs_2_AgBiBr_6_-Mo_2_CT_x_ (0.005 mg/mL) | 1.023 | 3.62 | 0.688 | 2.55 |
| Cs_2_AgBiBr_6_-Mo_2_CT_x_ (0.01 mg/mL) | 1.010 | 3.78 | 0.691 | 2.64 |
| Cs_2_AgBiBr_6_-Mo_2_CT_x_ (0.02 mg/mL) | 1.043 | 4.16 | 0.717 | 3.12 |
| Cs_2_AgBiBr_6_-Mo_2_CT_x_ (0.05 mg/mL) | 1.041 | 4.18 | 0.664 | 2.89 |

**Table S10.** Fitting parameters for EIS data.

| **Perovskite composition** | **R_1_ (Ω)** | **R_2_ (Ω)** |
| --- | --- | --- |
| Cs_2_AgBiBr_6_ | 101.4 | 1020.6 |
| Cs_2_AgBiBr_6_-V_2_CT_x_ | 78.7 | 357.3 |

# References

[1]. G. Kresse and J. Furthmüller, *Computational materials science*, **1996**, *6*, 15-50.

[2]. G. Kresse and D. Joubert, *Physical Review B*, **1999**, *59*, 1758.

[3]. J. P. Perdew, K. Burke and M. J. P. r. l. Ernzerhof, *Physical Review Letters*, **1996**, *77*, 3865.

[4]. S. Grimme, J. Antony, S. Ehrlich and H. Krieg, *Journal of Chemical Physics*, **2010**, *132*, 154104.

[5]. S. Grimme, S. Ehrlich and L. Goerigk, *Journal of Computational Chemistry*, **2011**, *32*, 1456-1465.

[6]. V. Wang, N. Xu, J.-C. Liu, G. Tang and W.-T. Geng, *J Computer Physics Communications*, **2021**, *267*, 108033.

[7]. K. Momma and F. Izumi, *J Applied Crystallography*, **2008**, *41*, 653-658.

[8]. J. Hutter, M. Iannuzzi, F. Schiffmann and J. VandeVondele, *Wiley Interdisciplinary Reviews: Computational Molecular Science*, **2014**, *4*, 15-25.
